# Ubiquitous [Na^+^]_i_/[K^+^]_i_-Sensitive Transcriptome in Mammalian Cells: Evidence for Ca^2+^_i_-Independent Excitation-Transcription Coupling

**DOI:** 10.1371/journal.pone.0038032

**Published:** 2012-05-29

**Authors:** Svetlana V. Koltsova, Yulia Trushina, Mounsif Haloui, Olga A. Akimova, Johanne Tremblay, Pavel Hamet, Sergei N. Orlov

**Affiliations:** 1 Centre de recherche, Centre hospitalier de l'Université de Montréal (CRCHUM) – Technopôle Angus, Montreal, PQ, Canada; 2 Institute of General Pathology and Pathophysiology, Russian Academy of Medical Sciences, Moscow, Russia; 3 Faculty of Biology, M.V. Lomonosov Moscow State University, Moscow, Russia; 4 Department of Medicine, Université de Montréal, Montreal, PQ, Canada; North Carolina State University, United States of America

## Abstract

Stimulus-dependent elevation of intracellular Ca^2+^ ([Ca^2+^]_i_) affects the expression of numerous genes – a phenomenon known as excitation-transcription coupling. Recently, we found that increases in [Na^+^]_i_ trigger *c-Fos* expression via a novel Ca^2+^_i_-independent pathway. In the present study, we identified ubiquitous and tissue-specific [Na^+^]_i_/[K^+^]_i_-sensitive transcriptomes by comparative analysis of differentially expressed genes in vascular smooth muscle cells from rat aorta (RVSMC), the human adenocarcinoma cell line HeLa, and human umbilical vein endothelial cells (HUVEC). To augment [Na^+^]_i_ and reduce [K^+^]_i_, cells were treated for 3 hrs with the Na^+^,K^+^-ATPase inhibitor ouabain or placed for the same time in the K^+^-free medium. Employing Affymetrix-based technology, we detected changes in expression levels of 684, 737 and 1839 transcripts in HeLa, HUVEC and RVSMC, respectively, that were highly correlated between two treatments (p<0.0001; R^2^>0.62). Among these Na^+^_i_/K^+^_i_-sensitive genes, 80 transcripts were common for all three types of cells. To establish if changes in gene expression are dependent on increases in [Ca^2+^]_i_, we performed identical experiments in Ca^2+^-free media supplemented with extracellular and intracellular Ca^2+^ chelators. Surprisingly, this procedure elevated rather than decreased the number of ubiquitous and cell-type specific Na^+^_i_/K^+^_i_-sensitive genes. Among the ubiquitous Na^+^_i_/K^+^_i_-sensitive genes whose expression was regulated independently of the presence of Ca^2+^ chelators by more than 3-fold, we discovered several transcription factors (*Fos, Jun, Hes1, Nfkbia*), interleukin-6, protein phosphatase 1 regulatory subunit, dual specificity phosphatase (*Dusp8*), prostaglandin-endoperoxide synthase 2, cyclin L1, whereas expression of metallopeptidase *Adamts1*, adrenomedulin, *Dups1, Dusp10* and *Dusp16* was detected exclusively in Ca^2+^-depleted cells. Overall, our findings indicate that Ca^2+^_i_-independent mechanisms of excitation-transcription coupling are involved in transcriptomic alterations triggered by elevation of the [Na^+^]_i_/[K^+^]_i_ ratio. There results likely have profound implications for normal and pathological regulation of mammalian cells, including sustained excitation of neuronal cells, intensive exercise and ischemia-triggered disorders.

## Introduction

Gene expression is regulated by diverse stimuli to achieve tissue-specific functional responses via coordinate synthesis of the cell's macromolecular components [1]. Electrochemical gradients of monovalent cations across the plasma membrane (high intracellular potassium, [K^+^]_i_ vs low intracellular sodium, [Na^+^]_i_) are created by the Na^+^,K^+^-pump and determine a large variety of physiologically important processes. These processes include maintenance of resting and action electrical membrane potentials, regulation of cell volume, secondary transport of mono- and divalent ions (such as chloride, calcium and phosphate), and accumulation of nutrients (glucose, amino acids, nucleotides) and other relevant molecules [2]. More recent studies demonstrated that side-by-side with the above-listed “classic” Na^+^_i_,K^+^_i_-dependent cellular processes, sustained elevation of the [Na^+^]_i_/[K^+^]_i_ ratio in vascular smooth muscle cells, cardiomyocytes, hepatocytes, renal epithelial and neuronal cells causes differential expression of *c-Fos* and other immediate response genes (IRG), as well as cell type-specific late response genes, such as tumour growth factor-β, the α1- and β1-subunits of Na^+^,K^+^-ATPase, myosin light chain, skeletal muscle actin, atrial natriuretic factor and mortalin (for review see [3]–[5]).

According to the generally accepted paradigm Na^+^_i_/K^+^_i_-sensitive mechanism of excitation-transcription coupling is driven by changes in intracellular [Ca^2+^] and activation of several Ca^2+^-sensitive pathways – a phenomenon termed excitation-transcription coupling [6]–[8]. Indeed, it is well-documented that elevation of the [Na^+^]_i_/[K^+^]_i_ ratio typically leads to increases in [Ca^2+^]_i_ via activation of the Na^+^/Ca^2+^ exchanger [9] and/or voltage-gated Ca^2+^ channels [10]. It has also been shown that promoters of numerous genes including *c-Fos* contain serum response element (SRE) and Ca^2+^+cAMP response element (CRE) activated by [Ca^2+^] increments in the cytoplasm and nucleus, respectively [11].

In contrast to the aforementioned mechanistic view, we found that in vascular smooth muscle cells from the rat aorta (RVSMC) and the human adenocarcinoma cell line (HeLa) the ouabain-induced changes in the c-Fos expression were preserved in the presence of Ca^2+^ channel blockers and extra- and intracellular Ca^2+^ chelators [12], [13]. These results made us conclude that along with canonical Ca^2+^_i_-mediated signaling, sustained elevation of the [Na^+^]_i_/[K^+^]_i_ ratio affects gene transcription via unknown Ca^2+^_i_-independent mechanism(s) [4]. In the present study, we deployed Affymetrix technology to characterize the relative impact of Ca^2+^_i_-mediated and -independent signaling on changes in gene expression triggered by sustained elevation of the [Na^+^]_i_/[K^+^]_i_ ratio. To accomplish this goal, we compared transcriptomes in 3 different cell types treated with 2 distinct Na^+^,K^+^-ATPase inhibitors in the absence and presence of Ca^2+^ chelators. Our results strongly indicate that, in mammalian cells, Ca^2+^_i_-independent pathways contribute to ubiquitous and cell type-specific transcriptomic alterations triggered by elevation of the [Na^+^]_i_/[K^+^]_i_ ratio.

## Results

### Effect of ouabain and K^+^-free medium on the [Na^+^]_i_/[K^+^]_i_ ratio and cell viability

**Figure 1:**
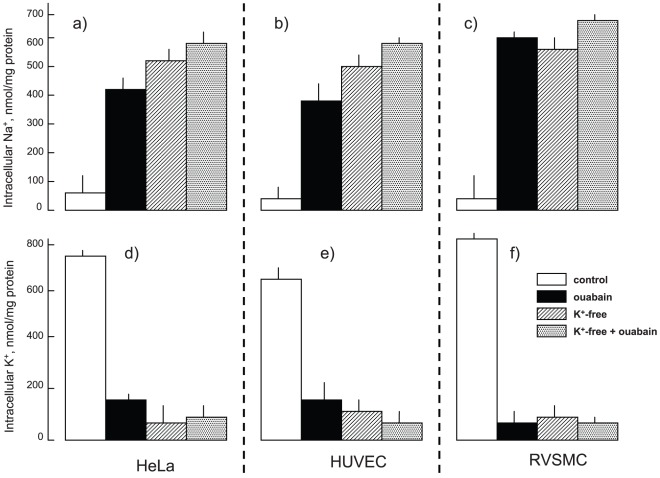
Effect of ouabain and K^+^-free medium on intracellular Na^+^ (a–c) and K^+^ (d–f) content in HeLa (a,d), HUVEC (b,e) and RVSMC (c,f). Cells were incubated in control or K^+^-free medium during 3 hr and ouabain was added at a final concentration of 3 µM (HeLa and HUVEC) or 3 mM (RVSMC). Mean ± S.E. values of experiments performed in quadruplicate are shown.

Figure 1 illustrates that 3-hr inhibition of the Na^+^,K^+^-ATPase by ouabain in HeLa, human umbilical vein endothelial cells (HUVEC) and RVSMC increased Na^+^_i_ content from ∼50 to 400–600 nmol/mg protein and decreased K^+^_i_ from 600–800 to ∼100 nmol/mg protein. In RVSMC the action of K^+^-free medium on the [Na^+^]_i_/[K^+^]_i_ ratio was similar, whereas in HUVEC and HeLa cells the gain of Na^+^_i_ and the loss of K^+^_i_ triggered by K^+^-free medium were higher compared to ouabain by ∼20% by ∼50%, respectively. Importantly, in all type of cells exposed to K^+^-free medium, the intracellular content of monovalent cations was not significantly affected by the addition of ouabain (Fig. 1). The control experiments demonstrated that 4 hr treatment with ouabain or K^+^-free medium as well as the addition of 50 µM EGTA and 10 µM BATPA-AM in Ca^2+^-free medium did not impact HeLa, HUVEC and RVSMC survival, as determined by lactate dehydrogenase (LDH) release, caspase-3 activity and chromatin cleavage assay (Table 1).

**Table 1:** Lactate dehydrogenase release (LDH, %), caspase-3 activity (CAS, nmol per mg of protein per hr) and chromatin cleavage (CHR, %) in cells treated with ouabain, K^+^-free and Ca^2+^-free medium during 4 hr.

Incubation medium, additions	HeLa			HUVEC			RVSMC		
	*LDH*	*CAS*	*CHR*	*LDH*	*CAS*	*CHR*	*LDH*	*CAS*	*CHR*
None (control)	5.3±1.6	0.36±0.05	3.3±0.9	7.0±2.1	0.26±0.07	4.4±0.7	3.0±0.6	0.46±0.04	5.1±1.2
Ouabain	6.3±0.6	0.44±0.07	2.9±1.6	7.3±1.6	0.41±0.08	6.9±1.8	4.3±1.1	0.37±0.03	3.9±2.6
K^+^-free medium	5.9±1.1	0.30±0.05	4.0±1.0	5.9±1.7	0.36±0.04	4.7±1.3	4.5±0.6	0.38±0.05	4.8±1.0
Ca^2+^-fre medium	6.9±1.2	0.46±0.09	4.9±1.6	8.5±3.0	0.53±0.07	7.1±1.7	5.5±1.1	0.57±0.08	6.9±2.2
Staurosporine	15.1±2.6*	1.33±0.25*	19.1±4.4*	35.3±3.9*	1.78±0.18*	32.1±6.4*	21.3±2.2*	2.91±0.30*	23.1±5.0*

To measure LDH release and chromatin cleavage, the total content of LDH and [^3^H]-labelled DNA were taken as 100%. Ouabain was added at final concentration of 3 µM (HeLa and HUVEC) or 3 mM (RVSMC). Ca^2+^-free medium contained 50 µM EGTA and 10 µM BAPTA-AM. Staurosporine, a potent trigger of apoptosis, was added as a positive control at concentration of 1 µM. Means ± S.E. from experiments performed with quadruplicate are given.

* p<0.05 as compared to controls.

### Effects of ouabain and K^+^-free medium on gene expression profile

Together with elevation of the [Na^+^]_i_/[K^+^]_i_ ratio, ouabain and K^+^-free medium may affect cells independently of suppression of Na^+^,K^+^-ATPase-mediated ion fluxes. Thus, recent studies have revealed that ouabain triggered interaction of the Na^+^,K^+^-ATPase α-subunit with the membrane-associated nonreceptor tyrosine kinase Src, activation of Ras/Raf/ERK1,2, phosphatidyl inositol 3-kinase (PI(3)K), PI(3)K-dependent protein kinase B, phospholipase C, [Ca^2+^]_i_ oscillations and augmented production of the reactive oxygen species (for review, see [14], [15]). On the other hand, the transfer of highly K^+^-permeable cells to K^+^-free medium results in transient membrane hyperpolarization, affecting the activity of diverse voltage-sensitive membrane-bound proteins [16], [17]. Considering this, we compared the actions of ouabain and K^+^-free medium on gene expression profiles in HUVEC, RVSMC and HeLa cells with a final goal of identifying ubiquitous and cell type-specific Na^+^_i_,K^+^_i_-sensitive transcriptomes.

**Figure 2:**
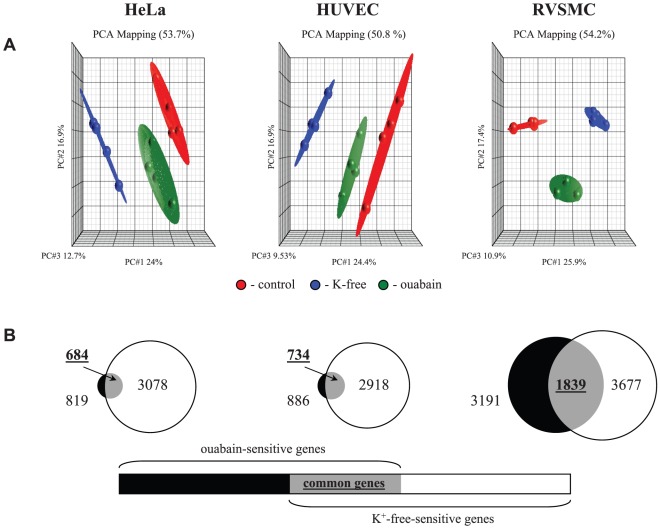
Comparative analysis of the actions of Na^+^,K^+^-ATPase inhibition by ouabain and K^+^-free medium on HeLa, HUVEC and RVSMC transcriptomes. **A.** Principal component analysis of the transcriptomes of HeLa, HUVEC and RVSMC. Cells were incubated for 3 hr in control Ca^2+^-containing media and processed for oligonucleotide microarray analysis as indicated in the Methods section. Ouabain was added at a final concentration of 3 µM (HeLa and HUVEC) or 3 mM (RVSMC). All experiments are repeated 4 times. Ellipsoids highlight portioning of samples based on the type of treatment. The principal components in 3-dimensional graphs (PC#1, PC#2 and PC#3) represent the variability of gene expression level within datasets. The total percentage of PCA mapping variability is shown on top. **B.** The total number of genes whose expression is altered by ouabain and K^+^-free medium by more than 1.2-fold with p<0.05 is indicated; numbers of genes affected by both stimuli appear in **bold**.

The data obtained in 4 independent experiments were normalized and then analyzed by principal component analysis (PCA) [18]. Each point on the PCA represents the gene expression profile of an individual sample. Samples that are near each other in the resulting 3-dimensional plot have a similar transcriptome while those that are further apart have dissimilar transcriptional profiles. This approach identified treatments with ouabain and K^+^-free medium as major sources of variability within datasets (Fig. 2A).

**Figure 3:**
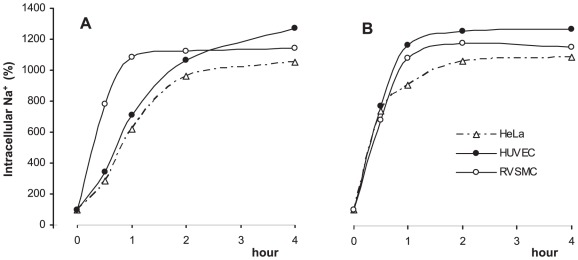
Kinetics of elevation of intracellular Na^+^ in HeLa, HUVEC and RVSMC triggered by ouabain (A) or K^+^-free medium (B). Ouabain was added at a final concentration of 3 µM (HeLa and HUVEC) or 3 mM (RVSMC). Intracellular Na^+^ content in the absence of Na^+^,K^+^-ATPase inhibitors was taken as 100%. Means obtained in experiments performed in triplicate are shown.

Figure 2B disclosed that the total numbers of differentially-expressed transcripts in HeLa, HUVEC and RVSMC treated for 3 hr with ouabain were 819, 886 and 3199, whereas inhibition of the Na^+^,K^+^-ATPase in K^+^-free medium altered the expression of 3078, 2858 and 3677 transcripts, respectively. In all types of cells, the number of up- and down regulated genes affected by these stimuli was about the same, whereas maximal fold of activation and suppression was 65 and 24, respectively (Table 2). The decreased number of differentially expressed transcripts detected in ouabain-treated HUVEC and HeLa cells, compared to RVSMC, can be explained by retarded kinetics of elevation of [Na^+^]_i_ in ouabain-treated human cells compared to ouabain-treated RVSMC (Fig. 3A) and cells subjected to Na^+^,K^+^-ATPase inhibition in K^+^-free medium (Fig. 3B). This observation is consistent with the slow kinetics of ouabain interaction with the human house-keeping α1 Na^+^,K^+^-ATPase isoform demonstrated in early investigations [19].

**Table 2:** Total numbers of differentially expressed transcripts in HeLa, HUVEC and RVSMC in 3-hr of Na^+^,K^+^-ATPase inhibition in control (Ca^2+^ containing) medium.

	Ouabain-treated cells	Cells treated with K^+^-free medium	Transcripts affected by both stimuli
**HeLa**			
***Up-regulated transcripts***			
Number of transcripts	338	1371	278
Maximal fold of activation	13.78	46.86	N.A.
***Down-regulated transcripts***			
Number of transcripts*	481	1707	406
Maximal fold of inhibition	2.85	7.96	N.A.
**HUVEC**			
***Up-regulated transcripts***			
Number of transcripts	400	1471	355
Maximal fold of activation	9.68	64.81	N.A.
***Down-regulated transcripts***			
Number of transcripts*	486	1447	379
Maximal fold of inhibition	2.40	5.33	N.A.
**RVSMC**			
***Up-regulated transcripts***			
Number of transcripts	1288	1872	894
Maximal fold of activation	9.46	24.32	N.A.
***Down-regulated transcripts***			
Number of transcripts*	1903	1805	945
Maximal fold of inhibition	4.15	7.50	N.A.

Transcripts whose expression was altered by more than 1.2-fold with p<0.05 were subjected to analysis. Ouabain was added at final concentration of 3 µM (HeLa and HUVEC) or 3 mM (RVSMC). N.A. – non-applicable.

**Figure 4:**
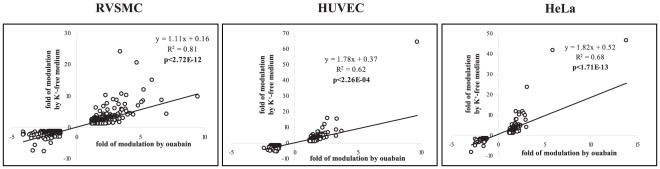
Correlation analysis of transcripts whose expression is altered by ouabain and K^+^-free medium in HeLa, HUVEC and RVSMC by more than by 1.2-fold with p<0.05. Cells were incubated during 3 hr; ouabain was added at a final concentration of 3 µM (HeLa and HUVEC) or 3 mM (RVSMC). Incubation medium contains 1.8 mM CaCl_2_. The total number of transcripts subjected to analysis is shown in Figure 2B. Transcript expression in control cells was taken as 1.00.

Further analysis determined that the expression of 684, 737 and 1839 transcripts in HeLa, HUVEC and RVSMC, respectively, was affected by both stimuli (Fig. 2B). Importantly, we observed highly significant (p<0.0003) and positive (R^2^>0.62) correlations between levels of differentially expressed transcripts identified in the presence of ouabain and K^+^-free medium (Fig. 4). Because the gain of Na^+^_i_ and loss of K^+^_i_ in cells treated with ouabain and K^+^-free medium are similar (Fig. 1), the results strongly suggest that the changes in gene expression evoked by both stimuli occur in response to elevation of the [Na^+^]_i_/[K^+^]_i_ ratio rather than due to Na^+^_i_,K^+^_i_-independent events. Considering this, we classified Na^+^_i_,K^+^_i_-sensitive transcriptomes as sets of genes whose expression was impacted by both ouabain and K^+^-free medium.

**Figure 5:**
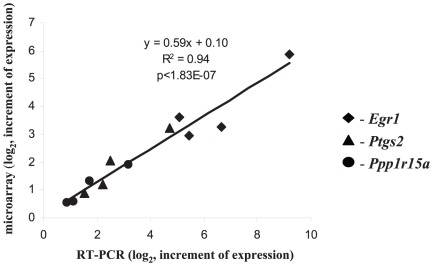
Verification of microarray results by quantitative RT-PCR. Gene expression was quantified for 3 hr incubation of control and Ca^2+^-depleted HUVEC in the presence of 3 µM ouabain or in K^+^-free medium. Mean values obtained in 4 independent experiments are shown. *Egr1* - early growth response protein 1, *Ptgs2* - prostaglandin-endoperoxide synthase 2, *Ppp1r15a* - protein phosphatase 1, regulatory (inhibitor) subunit 15A.

To confirm the ability of microarrays to resolve the differences in expression levels, we selected several Na^+^_i_/K^+^_i_-sensitive genes (*Egr1, Ptgs2* and *Ppp1r15a*) for additional validation by quantitative reverse transcription polymerase chain reaction (qRT-PCR) analysis. These experiments revealed highly significant correlations between the RT-PCR results and the validated microarray data (R^2^ = 0.94, p<0.0000002; Fig. 5).

### Ubiquitous Na^+^_i_,K^+^_i_-sensitive transcriptome

**Figure 6:**
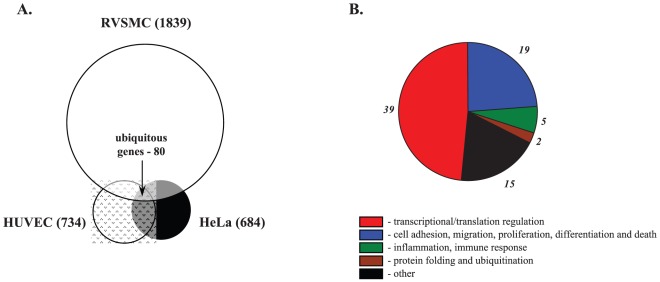
Na^+^_i_,K^+^_i_-sensitive transcriptomes identified in control Ca^2+^-containing media. **A.** Pie-chart showing the numbers of Na^+^_i_,K^+^_i_-sensitive genes detected in HeLa, HUVEC and RVSMC and ubiquitous Na^+^_i_,K^+^_i_-sensitive genes found in all 3 cell types. **B.** Distribution of ubiquitous Na^+^_i_,K^+^_i_-sensitive genes among major functional groups. Digitals shown in ***italics*** correspond to gene numbers for each functional group.

Among the Na^+^_i_,K^+^_i_-sensitive genes detected in HeLa, HUVEC and RVSMC, we identified 80 common genes, i.e. genes whose differential expression was increased or decreased by both ouabain and K^+^-free medium by at least 1.2-fold (p<0.05) in all 3 cell types (Fig. 6A). We noted that the list of ubiquitous Na^+^_i_,K^+^_i_-sensitive transcriptome was enriched with genes involved in the regulation of transcription/translation (49%), cell cycle, adhesion and migration (24%), and inflammatory and immune responses (6%) (Table 3, Fig. 6B).

**Table 3:** Ubiquitous Na^+^_i_/K^+^_i_-sensitive genes whose expression was up- and down-regulated by more than 1.2-fold (p≤0.05) in control (Ca^2+^-containing) medium.

No.	Gene symbol, title	RVSMC		HUVEC		HeLa	
		Fold of activation/inhibition		Fold of activation/inhibition		Fold of activation/inhibition	
		ouabain	K^+^-free medium	ouabain	K^+^-free medium	ouabain	K^+^-free medium
	***Up-regulated genes***						
**1_t_**	***Egr1*** *//early growth response 1*	**4.80**±0.18	**6.97**±0.18	**9.69**±0.15	**64.81**±0.12	**13.78**±0.15	**46.85**±0.09
**2_t_**	**Fos**//FBJ osteosarcoma oncogene	**3.17**±0.08	**8.76**±0.08	**2.60**±0.09	**15.97**±0.11	**5.88**±0.13	**41.93**±0.09
**3_t_**	**Fosb**//FBJ osteosarcoma oncogene B	**5.91**±0.11	**15.22**±0.12	**1.99**±0.08	**5.82**±0.10	**3.12**±0.16	**23.85**±0.11
**4_t_**	***Atf3*** *//activating transcription factor 3*	**4.72**±0.03	**20.63**±0.08	**1.86**±0.05	**7.50**±0.05	**2.28**±0.10	**11.29**±0.05
**5_t_**	**Zfp36**//zinc finger protein 36	**3.52**±0.10	**5.95**±0.09	**2.36**±0.10	**12.07**±0.11	**2.27**±0.04	**10.30**±0.10
**6_t_**	**Jun**//Jun oncogene	**1.28**±0.06	**1.62**±0.07	**1.94**±0.06	**5.11**±0.05	**1.91**±0.11	**8.03**±0.04
**7_i_**	**Il6**//interleukin 6	**3.40**±0.14	**4.76**±0.15	**1.44**±0.09	**2.12**±0.08	**2.01**±0.11	**8.01**±0.08
**8_d_**	**Ppp1r15a**//protein phosphatase 1, regulatory (inhibitor) subunit 1	**2.59**±0.12	**3.57**±0.09	**2.54**±0.10	**4.08**±0.10	**2.98**±0.16	**7.73**±0.14
**9_d_**	**Dusp8**//dual specificity phosphatase 8	**1.55**±0.11	**3.09**±0.12	**1.54**±0.08	**3.35**±0.06	**1.83**±0.11	**6.25**±0.10
**10_t_**	***Ddit3*** *//DNA-damage inducible transcript 3*	**2.15**±0.03	**4.26**±0.03	**1.35**±0.09	**4.91**±0.09	**1.52**±0.13	**5.46**±0.10
**11_t_**	***Junb*** *//jun B proto-oncogene*	**4.03**±0.08	**4.24**±0.07	**1.85**±0.09	**7.32**±0.07	**2.20**±0.09	**5.40**±0.08
**12_i_**	**Ptgs2**//prostaglandin-endoperoxide synthase 2	**9.46**±0.10	**9.95**±0.11	**3.73**±0.03	**7.84**±0.04	**2.39**±0.13	**5.15**±0.10
**13_t_**	***Cyr61*** *//cysteine-rich, angiogenic inducer, 61*	**1.71**±0.09	**2.00**±0.10	**1.34**±0.04	**2.11**±0.04	**2.02**±0.09	**5.11**±0.08
**14_t_**	***Nr4a2*** *//nuclear receptor subfamily 4, group A, member 2*	**5.19**±0.07	**8.65**±0.12	**2.03**±0.09	**3.94**±0.10	**2.39**±0.07	**4.76**±0.05
**15_i_**	**Nfkbiz**//nuclear factor of kappa light polypeptide gene enhance	**3.24**±0.09	**4.47**±0.08	**2.44**±0.06	**5.15**±0.02	**2.11**±0.07	**4.66**±0.07
**16_t_**	**Hes1**//hairy and enhancer of split 1 (Drosophila)	**3.00**±0.06	**6.15**±0.09	**2.07**±0.09	**5.86**±0.08	**1.71**±0.10	**4.62**±0.11
**17_d_**	***Spry4*** *//sprouty homolog 4 (Drosophila)*	**1.84**±0.06	**1.40**±0.09	**3.27**±0.12	**4.91**±0.12	**2.12**±0.17	**4.26**±0.17
**18_o_**	**Txnip**//thioredoxin interacting protein	**3.85**±0.10	**5.84**±0.10	**2.62**±0.09	**6.99**±0.12	**3.07**±0.10	**4.18**±0.09
**19_d_**	***Areg*** *//amphiregulin*	**1.90**±0.04	**1.75**±0.06	**1.36**±0.11	**1.39**±0.07	**1.93**±0.13	**4.13**±0.14
**20_i_**	**Nfkbia**//nuclear factor of kappa light polypeptide gene	**2.46**±0.03	**5.80**±0.05	**1.39**±0.09	**2.68**±0.08	**1.54**±0.10	**3.63**±0.10
**21_t_**	***Klf10*** *//Kruppel-like factor 10*	**2.80**±0.05	**3.66**±0.06	**1.83**±0.07	**4.53**±0.05	**1.74**±0.06	**3.57**±0.05
**22_d_**	**Plk3**//polo-like kinase 3 (Drosophila)	**3.29**±0.12	**3.64**±0.12	**1.22**±0.06	**2.15**±0.06	**1.41**±0.05	**2.83**±0.07
**23_d_**	**Ccnl1**//cyclin L1	**2.10**±0.06	**3.82**±0.08	**1.62**±0.05	**2.73**±0.04	**1.33**±0.09	**2.72**±0.08
**24_d_**	**Abl2**//v-abl Abelson murine leukemia viral oncogene homolog 2	**1.96**±0.11	**2.02**±0.08	**1.44**±0.06	**2.04**±0.05	**1.74**±0.07	**2.62**±0.05
**25_d_**	***Pmaip1*** *//phorbol-12-myristate-13-acetate-induced protein 1*	**1.46**±0.06	**1.64**±0.05	**1.51**±0.11	**2.30**±0.11	**1.71**±0.08	**2.55**±0.06
**26_t_**	***Bcl6*** *//B-cell CLL/lymphoma 6*	**1.64**±0.10	**1.88**±0.09	**1.89**±0.07	**3.78**±0.07	**1.32**±0.10	**2.53**±0.10
**27_t_**	***Mafk*** *//v-maf musculoaponeurotic fibrosarcoma oncogene homolog K*	**2.94**±0.08	**3.19**±0.11	**1.37**±0.08	**1.23**±0.08	**1.59**±0.07	**2.50**±0.07
**28_d_**	***Errfi1*** *//ERBB receptor feedback inhibitor 1*	**3.14**±0.07	**2.59**±0.10	**1.75**±0.07	**3.06**±0.07	**1.45**±0.05	**2.42**±0.06
**29_o_**	**Dusp16**//dual specificity phosphatase 16	**2.33**±0.08	**2.47**±0.09	**1.62**±0.09	**3.49**±0.08	**1.46**±0.11	**2.38**±0.11
**30_t_**	**Maff**//v-maf musculoaponeurotic fibrosarcoma oncogene homolog F	**2.47**±0.07	**3.11**±0.09	**1.27**±0.09	**2.25**±0.04	**1.42**±0.10	**2.35**±0.10
**31_t_**	***Tsc22d2*** *//TSC22 domain family, member 2*	**1.21**±0.04	**1.76**±0.06	**1.80**±0.05	**3.80**±0.03	**1.41**±0.05	**2.24**±0.05
**32_o_**	***Slc25a25*** *//solute carrier family 25*	**2.18**±0.03	**3.56**±0.05	**1.77**±0.06	**2.97**±0.05	**1.49**±0.05	**2.23**±0.07
**33_o_**	**Insig1**//insulin induced gene 1	**1.93**±0.08	**1.96**±0.09	**1.36**±0.04	**1.86**±0.03	**1.67**±0.06	**2.23**±0.05
**34_t_**	***Mxd1*** *//max dimerization protein 1*	**1.83**±0.06	**3.21**±0.05	**1.49**±0.07	**2.42**±0.06	**1.66**±0.08	**2.19**±0.08
**35_t_**	***Fosl1*** *//fos-like antigen 1*	**1.92**±0.07	**2.17**±0.07	**1.29**±0.04	**2.35**±0.04	**1.20**±0.04	**2.17**±0.06
**36_d_**	***Hbegf*** *//heparin-binding EGF-like growth factor*	**4.44**±0.14	**4.10**±0.18	**1.52**±0.06	**2.25**±0.03	**1.35**±0.07	**2.13**±0.08
**37_d_**	***Epha2*** *//Eph receptor A2*	**3.61**±0.08	**2.98**±0.12	**1.31**±0.07	**1.41**±0.06	**1.39**±0.11	**2.11**±0.08
**38_d_**	***Birc3*** *//baculoviral IAP repeat-containing 3*	**3.30**±0.03	**7.47**±0.03	**1.69**±0.09	**4.58**±0.12	**1.29/**±0.09	**2.04**±0.09
**39_d_**	***Efna1*** *//ephrin A1*	**1.82**±0.04	**1.26**±0.03	**1.48**±0.04	**1.83**±0.05	**1.34**±0.09	**2.02**±0.09
**40_t_**	***Zc3h12c*** *//zinc finger CCCH type containing 12C*	**1.39**±0.06	**1.70**±0.02	**1.76**±0.09	**1.62**±0.09	**1.68**±0.08	**2.01**±0.07
**41_o_**	***Ldlr*** *//low density lipoprotein receptor*	**1.50**±0.04	**1.51**±0.04	**1.48**±0.05	**1.86**±0.05	**1.75**±0.03	**1.95**±0.04
**42_t_**	***Sertad2*** *//SERTA domain containing 2*	**1.74**±0.06	**1.60**±0.07	**1.36**±0.08	**1.79**±0.07	**1.29**±0.09	**1.93**±0.07
**43_t_**	***Zc3h12a*** *//zinc finger CCCH type containing 12A*	**2.14**±0.06	**2.25**±0.08	**1.35**±0.06	**2.68**±0.07	**1.25**±0.05	**1.92**±0.03
**44_t_**	***Cpeb4*** *//cytoplasmic polyadenylation element binding protein 4*	**2.46**±0.04	**2.59**±0.05	**1.26**±0.06	**1.74**±0.07	**1.31**±0.06	**1.87**±0.05
**45_i_**	***Il1rap*** *//interleukin 1 receptor accessory protein*	**1.32**±0.09	**1.21**±0.06	**1.33**±0.10	**1.99**±0.10	**1.42**±0.09	**1.71**±0.07
**46_f_**	***Dnajb9*** *//DnaJ (Hsp40) homolog, subfamily B, member 9*	**1.33**±0.03	**1.30**±0.03	**1.63**±0.05	**2.20**±0.06	**1.46**±0.07	**1.67**±0.10
**47_t_**	***Ppp1r15b*** *//protein phosphatase 1*	**1.72**±0.02	**1.77**±0.02	**1.30**±0.05	**1.63**±0.03	**1.35**±0.04	**1.66**±0.03
**48_d_**	***Dusp6*** *//dual specificity phosphatase 6*	**3.36**±0.10	**1.73**±0.11	**1.47**±0.12	**1.81**±0.09	**1.48**±0.07	**1.64**±0.07
**49_d_**	***Zswim6*** *//zinc finger, SWIM-type containing 6*	**1.44**±0.03	**1.70**±0.04	**1.22**±0.03	**1.64**±0.03	**1.35**±0.02	**1.62**±0.03
**50_t_**	***Zbtb43*** *//zinc finger and BTB domain containing 43*	**1.34**±0.05	**2.14**±0.05	**1.24**±0.06	**1.76**±0.08	**1.39**±0.05	**1.59**±0.05
**51_o_**	***Slc20a1*** *//solute carrier family 20 (phosphate transporter)*	**1.56**±0.04	**1.29**±0.04	**1.30**±0.03	**1.43**±0.04	**1.29**±0.04	**1.56**±0.04
**52_o_**	***Hmgcs1*** *//3-hydroxy-3-methylglutaryl-Coenzyme A synthase 1*	**1.80**±0.03	**2.01**±0.03	**1.24**±0.02	**1.56**±0.01	**1.38**±0.04	**1.44**±0.03
**53_f_**	***Coq10b*** *//coenzyme Q10 homolog B (S. cerevisiae)*	**1.71**±0.03	**1.47**±0.03	**1.22**±0.04	**1.47**±0.05	**1.41**±0.05	**1.43**±0.06
**54_t_**	***Clk1*** *//CDC-like kinase 1*	**1.37**±0.07	**2.14**±0.07	**1.40**±0.07	**1.66**±0.07	**1.41**±0.06	**1.40**±0.07
**55_t_**	***Zbtb11*** *//zinc finger and BTB domain containing 11*	**2.16**±0.05	**2.00**±0.08	**1.42**±0.05	**1.51**±0.05	**1.31**±0.04	**1.36**±0.05
**56_t_**	***Nr1d1*** *//nuclear receptor subfamily 1, group D, member 1*	**1.57**±0.05	**1.74**±0.08	**1.43**±0.10	**1.48**±0.07	**1.50**±0.07	**1.31**±0.09
**57_o_**	***Stard4*** *//StAR-related lipid transfer (START) domain*	**1.30**±0.05	**1.51**±0.05	**1.32**±0.06	**1.46**±0.03	**1.39**±0.07	**1.30**±0.05
**58_t_**	**Nfya**//nuclear transcription factor-Y alpha	**1.52**±0.06	**1.45**±0.09	**1.49**±0.04	**1.48**±0.04	**1.41**±0.05	**1.30**±0.04
**59_t_**	***Jmjd1c*** *//jumonji domain containing 1C*	**1.49**±0.03	**1.59**±0.05	**1.39**±0.03	**1.52**±0.05	**1.28**±0.04	**1.29**±0.05
**60_t_**	***Dcp1a*** *//DCP1 decapping enzyme homolog A (S. cerevisiae)*	**1.66**±0.02	**1.65**±0.06	**1.24**±0.03	**1.26**±0.03	**1.27**±0.06	**1.28**±0.04
**61_t_**	***E2f3*** *//E2F transcription factor 3*	**1.26**±0.06	**1.43**±0.05	**1.41**±0.08	**1.73**±0.08	**1.22**±0.06	**1.27**±0.04
	***Down-regulated genes***						
**1_o_**	***Gabre*** *//gamma-aminobutyric acid (GABA) A receptor, epsilon*	**−1.21**±0.08	**−1.45**±0.06	**−1.33**±0.06	**−1.48**±0.03	**−2.01**±0.09	**−3.15**±0.14
**2_t_**	**Hoxb5**//homeo box B5	**−2.25**±0.12	**−2.82**±0.13	**−1.75**±0.06	**−3.00**±0.08	**−1.49**±0.08	**−2.35**±0.09
**3_t_**	***Znf250*** *//zinc finger protein 250*	**−1.26**±0.07	**−1.20**±0.08	**−1.62**±0.06	**−1.90**±0.05	**−1.72**±0.07	**−2.08**±0.06
**4_t_**	**Rpp40**//ribonuclease P 40 subunit (human)	**−1.70**±0.08	**−1.84**±0.05	**−1.41**±0.03	**−1.79**±0.06	**−1.27**±0.05	**−2.03**±0.06
**5_t_**	***Rbm45*** *//RNA binding motif protein 45*	**−1.33**±0.05	**−1.37**±0.05	**−1.41**±0.06	**−1.67**±0.05	**−1.48**±0.11	**−1.89**±0.08
**6_d_**	***Aggf1*** *//angiogenic factor with G patch and FHA domains 1*	**−1.54**±0.04	**−1.24**±0.06	**−1.30**±0.08	**−1.29**±0.08	**−1.54**±0.07	**−1.83**±0.06
**7_d_**	***Fancf*** *//Fanconi anemia, complementation group F*	**−1.51**±0.07	**−1.30**±0.06	**−1.85**±0.08	**−1.71**±0.07	**−1.48**±0.10	**−1.83**±0.09
**8_o_**	***Rhobtb1*** *//Rho-related BTB domain containing 1*	**−1.98**±0.06	**−1.35**±0.06	**−1.41**±0.08	**−1.48**±0.08	**−1.34**±0.11	**−1.80**±0.07
**9_t_**	***Znf691*** *//zinc finger protein 691*	**−1.42**±0.09	**−1.33**±0.07	**−1.28**±0.08	**−1.23**±0.07	**−1.46**±0.11	**−1.67**±0.06
**10_o_**	***Rrs1*** *//RRS1 ribosome biogenesis regulator homolog (S. cerevisiae)*	**−1.26**±0.07	**−1.39**±0.10	**−1.31**±0.08	**−1.33**±0.07	**−1.29**±0.03	**−1.59**±0.03
**11_t_**	**Mrpl46**//mitochondrial ribosomal protein L46	**−1.34**±0.04	**−1.24**±0.05	**−1.21**±0.04	**−1.34**±0.03	**−1.24**±0.05	**−1.58**±0.07
**12_o_**	***Golga5*** *//golgi autoantigen, golgin subfamily a*	**−1.65**±0.04	**−1.21**±0.06	**−1.38**±0.05	**−1.48**±0.05	**−1.29**±0.05	**−1.56**±0.04
**13_d_**	***Psrc1*** *//proline/serine-rich coiled-coil 1*	**−1.65**±0.08	**−1.80**±0.07	**−1.25**±0.08	**−1.44**±0.05	**−1.23**±0.04	**−1.53**±0.05
**14_o_**	***Spata7*** *//spermatogenesis associated 7/*	**−1.55**±0.07	**−1.32**±0.06	**−1.29**±0.06	**−1.31**±0.06	**−1.38**±0.08	**−1.49**±0.08
**15_d_**	***Tgfbrap1*** *//transforming growth factor, beta receptor*	**−1.49**±0.06	**−1.22**±0.05	**−1.36**±0.05	**−1.55**±0.03	**−1.29**±0.06	**−1.47**±0.05
**16_o_**	***Hps6*** *//Hermansky-Pudlak syndrome 6*	**−2.15**±0.07	**−1.69**±0.10	**−1.39**±0.09	**−1.31**±0.10	**−1.48**±0.08	**−1.46**±0.06
**17_t_**	***Znf18*** *4//zinc finger protein 184*	**−1.78**±0.06	**−1.55**±0.08	**−1.71**±0.10	**−2.01**±0.08	**−1.28**±0.07	**−1.44**±0.06
**18_t_**	***Pars2*** *//prolyl-tRNA synthetase (mitochondrial)(putative)*	**−1.61**±0.04	**−1.20**±0.05	**−1.28**±0.05	**−1.28**±0.06	**−1.38**±0.05	**−1.34**±0.04
**19_o_**	***Sh3bp5l*** *//SH3 binding domain protein 5 like*	**−1.82**±0.04	**−1.54**±0.03	**−1.46**±0.07	**−1.40**±0.09	**−1.35**±0.07	**−1.27**±0.07

HeLa, HUVEC and RVSMC were treated with ouabain or K+-free medium for 3 hr. Genes whose expression is not affected by Na^+^,K^+^-ATPase inhibition at least in one type of Ca^2+^-depleted cells are shown in ***italics***. Genes whose expression is not affected by Na^+^,K^+^-ATPase inhibition in all 3 types of cells are shown in ***underlined italics***. Functional categories are indicated in the left column as: **t** – regulators of transcription/translation, RNA processing and degradation; **d** – regulators of cell adhesion, migration, proliferation, differentiation and death; **f** - protein folding and ubiquitination; **i** – inflammation and immune response; **o** – other functional categories and genes with unknown function.

Ubiquitous Na^+^_i_/K^+^_i_-sensitive genes, whose expression was increased by more than 3-fold, included the transcriptional regulator of C2H2-type zinc-finger protein *Egr-1*, members of the superfamily of b-zip transcriptional factors possessing basic DNA-binding domain and leucine-zipper dimerization motif and forming heterodimeric activating protein-1 (AP-1) (*Fos, FosB, Jun, JunB, Atf3*), transcription factor of the steroid-thyroid hormone-retinoid receptor superfamily *Nr4a2* and the basic helix-loop-helix transcription regulator *Hes1*. *Nfkbiz* and *Nfkbia* are transcriptional regulators of genes encoding intermediates of inflammation whereas interleukin 6 (*Il6*) is a potent controller of the acute inflammatory response phase. Prostaglandin-endoperoxide synthase 2 (*Ptgs2*) also known as cyclooxygenase-2 is a key enzyme in the biosynthesis of prostaglandins implicated in inflammatory responses and mitogenesis. Na^+^_i_/K^+^_i_-sensitive regulators of cell proliferation, differentiation and death whose expression is sharply increased are represented by dual specificity protein phosphatase *Dusp8*, inhibitor of the receptor-transduced mitogen-activated protein kinase signaling pathway *Spry4*, the protein phosphatase 1 regulatory subunit *Ppp1r15a*, the cytokine inducible kinase *Plk3*, a member of the epidermal growth factor (EGF) family amphiregulin (*Areg*), and heparin-binding EGF-like growth factor *Hbegf*.

Among ubiquitous Na^+^_i_,K^+^_i_-sensitive genes from other functional categories, we noted augmented expression of oxidative stress mediator *Txnip*, the low-density lipoprotein receptor *Ldlr*, the regulator of cholesterol synthesis *Insig1* and 2 carriers involved in intracellular phosphate handling, i.e. the Ca^2+^-dependent mitochondrial solute carrier *Slc25a25* mediating ATP-Mg/P_i_ exchange and the sodium-phosphate symporter *Slc20a1* (Table 3). In contrast to substantial number of ubiquitous Na^+^_i_/K^+^_i_-sensitive genes that were strongly up-regulated, we identified only one transcript – the epsilon subunit of GABA-activated Cl^−^ channel (*Gabre*) – whose expression was decreased by more than 3-fold (Table 3).

### Cell type-specific Na^+^_i_,K^+^_i_-sensitive transcriptomes

Because the number of cell-type specific Na^+^_i_,K^+^_i_-sensitive transcripts is very large (Fig. 2B, Table 2), we restricted their functional characterization to genes whose expression was altered by more than 4-fold. The relevant analysis led us to several conclusions. *First*, ubiquitous genes comprised up to ∼50% of Na^+^_i_,K^+^_i_-sensitive genes whose expression was increased in HeLa, HUVEC and RVSMC by more than 4-fold (Tables 4,5,6). *Second*, strongly up-regulated Na^+^_i_,K^+^_i_-sensitive genes are abundant with the transcripts that were detected in 2 cell types. These transcripts (underlined and **bold**) accounted for 37%, 18% and 16% of the total numbers of genes that manifested up- and down-regulation in HeLa, HUVEC and RVSMC, respectively. *Third*, similar to ubiquitously regulated genes (Table 3), the list of cell type-specific Na^+^_i_,K^+^_i_-sensitive genes that were strongly up- and down-regulated was enriched with the transcriptional regulators and regulators of immune responses and inflammation. Examples included nuclear receptor subfamily 4 group A *Nr4a1* and *Nr4a3* in HeLa and RVSMC, early growth response 2 *Egr2* in HeLa and RVSMC, immediate early response 2 *Ier2* in RVSMC, interleukin 8 *IL8* in HeLa and HUVEC, tumour necrosis factor α-induced protein 3 *TNFAIP3* in HeLa and HUVEC. From other functional categories, we observed substantial cell type-specific elevation of colony stimulating factor 3 *Csf3* in RVSMC, metallopeptidase *ADAMTS1* and vasoactive intestinal peptide *VIP* in HUVEC, the regulator of G-protein signalling *Rgs2* in RVSMC, endothelin 1 *EDN1* and the inhibitor of DNA binding-2 *ID2* in HeLa cells (Tables 4,5,6).

**Table 4:** ***HeLa cells*** **:** the list of genes whose expression was changed by more than 4-fold in 3 hr of Na^+^,K^+^-ATPase inhibition in control (Ca^2+^ containing) medium.

Gene symbol, title	Affymetrix ID	Fold of activation or inhibition (-) by ouabain/p value	Fold of activation or inhibition (-) by K^+^-free medium/p value	Fold of activation by ouabain/K^+^-free medium in HUVEC or RVSMC
EGR1//early growth response 1	8108370	**13.78/**7.27E-08	**46.85/**8.18E-05	
FOS//FBJ murine osteosarcoma viral oncogene	7975779	**5.88/**7.27E-08	**41.93/**8.56E-06	
FOSB//FBJ murine osteosarcoma viral oncogene	8029693	**3.12/**2.78E-07	**23.85/**1.51E-05	
**NR4A1**//nuclear receptor subfamily 4, group A,	7955589	**2.63/**1.39E-07	**11.79/**8.56E-06	3.52/10.73 **(HUVEC)**
**TNFAIP3**//tumor necrosis factor, alpha-induced protein 3	8122265	**1.90/**2.27E-07	**11.77/**8.56E-06	3.34/15.71 **(HUVEC)**
ATF3//activating transcription factor 3	7909610	**2.28/**9.86E-08	**11.29/**8.56E-06	
**EDN1**//endothelin 1	8116921	**2.31/**2.55E-06	**11.15/**1.10E-04	
**NR4A3**//nuclear receptor subfamily 4, group A	8156848	**2.77/**8.28E-08	**11.06/**8.56E-06	4.16/4.68 **(HUVEC)**
ZFP36//zinc finger protein 36, C3H type, homolog (mouse)	8028652	**2.27/**9.86E-08	**10.30/**8.56E-06	
**IL8**//interleukin 8	8095680	**2.93/**5.07E-06	**10.00/**5.31E-04	1.96/4.45 **(HUVEC)**
JUN//jun oncogene	7916609	**1.91/**1.68E-07	**8.03/**8.56E-06	
IL6//interleukin 6 (interferon, beta 2)	8131803	**2.01/**3.12E-07	**8.01/**1.51E-05	
PPP1R15A//protein phosphatase 1, regulatory (inhibitor) subunit 15A	8030128	**2.98/**2.08E-06	**7.73/**5.19E-04	
DUSP8//dual specificity phosphatase 8	7945641	**1.83/**4.85E-07	**6.25/**2.05E-05	
**EGR2**//early growth response 2	7933872	**1.85/**1.39E-06	**5.79/**6.41E-05	2.87/9.08 **(RVSMC)**
DDIT3//DNA-damage-inducible transcript 3	7964460	**1.52/**2.19E-06	**5.46/**4.39E-05	
JUNB//jun B proto-oncogene	8026047	**2.20/**5.35E-07	**5.40/**7.13E-05	
PTGS2//prostaglandin-endoperoxide synthase 2	7922976	**2.39/**4.30E-06	**5.15/**9.30E-04	
CYR61//cysteine-rich, angiogenic inducer, 61	7902687	**2.02/**2.78E-07	**5.11/**2.71E-05	
CSRNP1//cysteine-serine-rich nuclear protein 1	8086330	**2.32/**2.88E-06	**4.80/**7.31E-04	
**HIST1H3J**//histone cluster 1, H3j	8124537	**1.69/**2.25E-05	**4.77/**6.25E-04	1.40/1.62 **(HUVEC)**
NR4A2//nuclear receptor subfamily 4, group A,	8055952	**2.39/**1.39E-07	**4.76/**4.04E-05	
NFKBIZ//nuclear factor of kappa light polypeptide gene enhancer in B-cells inhibitor, zeta	8081386	**2.11/**3.12E-07	**4.66/**5.06E-05	
AREG//amphiregulin	8095744	**2.10/**2.01E-05	**4.64/**2.04E-03	
**DUSP1**//dual specificity phosphatase 1	8115831	**2.24/**1.60E-07	**4.64/**4.09E-05	1.59/3.81 **(HUVEC)**
HES1//hairy and enhancer of split 1, (Drosophila)	8084880	**1.71/**1.05E-06	4.62/4.57E-05	
SPRY4//sprouty homolog 4 (Drosophila)	8114797	**2.12/**3.80E-05	**4.26/**5.65E-03	
TXNIP//thioredoxin interacting protein	7904726	**3.07/**6.87E-07	**4.18/**1.03E-02	
**DUSP10**//dual specificity phosphatase 10	7924450	**1.88/**3.99E-07	**4.04/**4.71E-05	1.32/3.02 **(HUVEC)**
**CDKN2AIP**//CDKN2A interacting protein	8098500	**1.84/**2.19E-06	**4.01/**2.10E-04	1.26/2.05 **(HUVEC)**
**ID2**//inhibitor of DNA binding 2, dominant negative helix-loop-helix protein	8040103	**1.64/**2.91E-06	**4.01/**1.25E-04	
**LOC100287934/**/similar to hCG2042721	7909990	**−2.41/**2.12E-03	**−4.01/**3.18E-05	−1.96/−4.68 **(HUVEC)**
**LOC100131860**//hypothetical protein	7991047	**−2.47/**3.26E-03	**−4.73/**3.32E-05	
**TRIM52**//tripartite motif-containing 52	8110666	**−1.57/**6.01E-03	**−5.03/**8.88E-07	−1.64/−3.85 **(HUVEC)**
**C9orf3**//chromosome 9 open reading frame 3	8156571	**−2.85/**4.40E-05	**−7.96/**1.39E-07	−1.71/−2.24 **(HUVEC)**

Genes whose differential expression is limited to HeLa cells are shown in **bold**. Genes whose differential expression were also detected in HUVEC or RVSMC are shown in **underlined bold**. All transcripts listed in this table were also differentially expressed in Ca^2+^-depleted cells.

**Table 5:** ***HUVEC:*** the list of genes whose expression was changed in by more than 4-fold in 3 hr of Na^+^,K^+^-ATPase inhibition in control (Ca^2+^ containing) medium.

Gene symbol, title	Affymetrix ID	Fold of activation or inhibition (-) by ouabain/p value	Fold of activation or inhibition (-) by K^+^-free medium/p value	Fold of modulation by ouabain/K^+^-free medium in HeLa or RVSMC
EGR1//early growth response 1	8108370	**9.69**/1.10E-05	64.81/1.98E-08	
FOS//FBJ murine osteosarcoma viral oncogene	7975779	**2.60**/2.41E-04	15.97/7.24E-08	
**TNFAIP3**//tumor necrosis factor, alpha-induced protein 3	8122265	**3.34**/7.23E-05	15.71/7.53E-08	1.90/11.77 **(HeLa)**
ZFP36//zinc finger protein 36, C3H type,	8028652	**2.36**/5.36E-04	12.07/1.99E-07	
**NUAK2**//NUAK family, SNF1-like kinase, 2	7923753	2.22/3.28E-04	9.97/9.48E-08	
**IL1A**//interleukin 1, alpha	8054712	3.30/5.79E-05	8.88/2.11E-07	
**CD274**//CD274 molecule	8154233	3.71/1.71E-05	7.96/8.93E-08	1.42/2.31**(HeLa)**
PTGS2//prostaglandin-endoperoxide synthase 2	7922976	3.73/6.24E-08	7.84/1.12E-09	
ATF3//activating transcription factor 3	7909610	1.86/7.23E-05	7.50/1.46E-08	
JUNB//jun B proto-oncogene	8026047	1.85/6.08E-04	7.32/1.26E-07	
TXNIP//thioredoxin interacting protein	7904726	2.62/3.51E-04	6.99/7.32E-07	
**ADAMTS1**//ADAM metallopeptidase with thrombospondin type 1 motif, 1	8069676	2.42/2.49E-04	6.31/4.46E-07	
est (ncrna:misc_RNA)	7919749	2.20/4.60E-03	6.23/7.44E-06	
HES1//hairy and enhancer of split 1, (Drosophila)	8084880	2.07/2.77E-04	5.86/2.11E-07	
**KITLG**//KIT ligand	7965322	1.85/4.92E-05	5.85/1.70E-08	
FOSB//FBJ murine osteosarcoma viral oncogene	8029693	1.99/6.50E-04	5.82/4.46E-07	
Novel scRNA pseudogene	8160086	1.47/2.70E-02	5.64/1.49E-06	
**SNAPC1**//small nuclear RNA activating complex, polypeptide 1, 43 kD	7974870	2.17/2.13E-05	5.48/1.98E-08	
NFKBIZ//nuclear factor of kappa light polypeptide gene enhancer in B-cells inhibitor, zeta	8081386	2.44/1.10E-05	5.15/2.16E-08	
JUN//jun oncogene	7916609	1.94/3.43E-05	5.11/1.98E-08	
**FAM148B**//family with sequence similarity 148, member B	7989473	2.28/4.60E-03	5.09/2.05E-05	
**CXCL1**//chemokine (C-X-C motif) ligand 1	8095697	2.03/2.26E-03	5.02/3.75E-06	**2.97/4.02 (RVSMC)**
SPRY4//sprouty homolog 4 (Drosophila)	8114797	3.27/5.05E-05	4.91/9.53E-07	
DDIT3//DNA-damage-inducible transcript 3	7964460	1.35/1.12E-02	4.91/2.20E-07	
**HSPA1B**//heat shock 70 kDa protein 1B	8178086	1.81/1.22E-02	4.78/1.22E-05	
BIRC3//baculoviral IAP repeat-containing 3	7943413	1.69/5.91E-03	4.58/2.71E-06	
KLF10//Kruppel-like factor 10	8152215	1.83/2.41E-04	4.53/1.34E-07	
**IL8**//interleukin 8	8095680	1.96/2.61E-03	4.45/4.97E-06	2.93/10.00 **(HeLa)**
*est (ncrna:snoRNA)*	*8126093*	*1.88/4.07E-04*	*4.41/4.46E-07*	
**HSPA1A**//heat shock 70 kDa protein 1A	8118310	1.85/7.33E-03	4.39/1.10E-05	
SNORD3A//small nucleolar RNA, C/D box 3A	8005547	1.29/2.70E-02	4.31/4.46E-07	
est (ncrna:misc_RNA)	8122816	1.52/8.96E-03	4.30/1.59E-06	
PPP1R15A//protein phosphatase 1, regulatory (inhibitor) subunit 15A	8030128	2.54/1.58E-04	4.08/1.26E-06	
**VIP**//vasoactive intestinal peptide	8122865	1.53/4.54E-02	4.02/1.97E-05	
**KDM6B**//lysine (K)-specific demethylase 6B	8004671	1.63/5.12E-03	4.01/2.53E-06	1.73/2.83 **(HeLa)**
**SNORD52**//small nucleolar RNA, C/D box 52	8118322	−2.12/5.04E-03	−4.14/2.96E-05	
**est** (ncrna:snoRNA)	8012906	−1.94/5.91E-04	−4.39/8.68E-07	
**LOC100287934**//similar to hCG2042721	7896754	−1.93/2.58E-03	−4.46/4.31E-06	−2.41/−4.01 **(HeLa)**
**est** (ncrna:misc_RNA)	8007990	−1.37/4.92E-02	−5.03/1.59E-06	

Genes whose differential expression is limited to HUVEC are shown in **bold**. Genes whose expression were also detected in HeLa or RVSMC are shown in **underlined bold**. Genes whose expression was not affected by Na^+^,K^+^-ATPase inhibition in Ca^2+^-depleted cells are shown in *italics*.

**Table 6:** ***RVSMC:*** the list of genes whose expression was changed by more than 4-fold in 3 hr of Na^+^,K^+^-ATPase inhibition in control (Ca^2+^ containing) medium.

Gene symbol, title	Affymetrix ID	Fold of activation or inhibition (-) by ouabain/p value	Fold of activation or inhibition (-) by K^+^-free medium/p value	Fold of modulation by ouabain/K^+^-free medium in HUVEC or HeLa
**Cxcl2**//chemokine (C-X-C motif) ligand 2	10775896	**3.44/**7.21E-07	**24.32/**1.69E-09	1.38/2.58 **(HUVEC)**
*Atf3//activating transcription factor 3*	*10770710*	***4.72/*** *2.24E-07*	***20.63/*** *2.72E-09*	
Fosb//FBJ osteosarcoma oncogene B	10719432	**5.91/**1.15E-06	**15.22/**4.45E-08	
**Btg2**//B-cell translocation gene 2, anti-proliferative	10767767	**5.28/**1.08E-05	**12.02/**6.67E-07	
**Nr4a1**//nuclear receptor subfamily 4, group A	10899387	**3.52/**2.89E-05	**10.73/**4.79E-07	2.63/11.79 **(HeLa)**
*Csf2//colony stimulating factor 2*	*10742663*	***2.26/*** *2.74E-05*	***10.35/*** *4.23E-08*	
**cDNA**:known	10708091	**3.89/**1.50E-07	**10.31/**3.10E-09	
Ptgs2//prostaglandin-endoperoxide synthase 2	10764551	**9.46/**1.08E-07	**9.95/**3.97E-08	
***Egr2*** *//early growth response 2*	*10832802*	***2.87/*** *2.64E-06*	***9.08/*** *2.01E-08*	***1.85/5.79 (HeLa)***
Fos//FBJ osteosarcoma oncogene	10886031	**3.17/**3.92E-06	**8.76/**5.28E-08	
***Lif*** *//leukemia inhibitory factor*	*10773853*	***6.68/*** *7.21E-07*	***8.68/*** *1.47E-07*	
Nr4a2//nuclear receptor subfamily 4, group A	10845384	**5.19/**1.51E-06	**8.65/**1.47E-07	
**Axud1**//AXIN1 up-regulated 1	10920967	**5.10/**7.51E-08	**8.22/**4.42E-09	
**Rgs2**//regulator of G-protein signaling 2	10768332	**2.41/**6.15E-04	**7.67/**3.13E-06	1.31/2.65 **(HUVEC)**
**Snf1lk**//SNF1-like kinase	10832197	**3.80/**4.77E-06	**7.55/**2.42E-07	
Birc3//baculoviral IAP repeat-containing 3	10914799	**3.30/**2.04E-08	**7.47/**3.66E-10	
*Egr1//early growth response 1*	*10800919*	***4.80/*** *8.98E-06*	***6.97/*** *1.76E-06*	
*Plk2//polo-like kinase 2 (Drosophila)*	*10812954*	***2.14/*** *1.27E-06*	***6.77*** *3.10E-09*	
**Irf1**//interferon regulatory factor 1	10733553	**1.51/**1.47E-04	**6.33/**1.70E-08	
Hes1//hairy and enhancer of split 1	10754943	**3.00/**2.07E-06	**6.15/**5.28E-08	
**Egr3**//early growth response 3	10781337	**3.87/**4.79E-07	**6.00/**4.45E-08	
Zfp36//zinc finger protein 36	10720215	**3.52/**1.19E-06	**5.95/**7.06E-08	
Txnip//thioredoxin interacting protein	10817552	**3.85/**1.47E-06	**5.84/**1.46E-07	
Nfkbia//nuclear factor of kappa light polypeptide gene enhancer in B-cells inhibitor, alpha	10890024	**2.46/**3.55E-07	**5.80/**3.36E-09	
**Cish**//cytokine inducible SH2-containing protein	10912908	**3.59/**8.97E-08	**5.76/**4.42E-09	
**Ier2**//immediate early response 2	10806685	**3.88/**1.82E-06	**5.26/**2.80E-07	
**Hand1**//heart and neural crest derivatives	10719358	**1.86/**7.51E-03	**5.16/**1.95E-05	
Il6//interleukin 6	10859799	**3.40/**1.21E-05	**4.76/**2.03E-06	
**Nr4a3**//nuclear receptor subfamily 4, group A	10868940	**4.16/**1.99E-05	**4.68/**9.44E-06	**2.77/11.06 (HeLa)**
Nfkbiz//nuclear factor of kappa light polypeptide gene enhancer in B-cells inhibitor, zeta	10750848	**3.24/**8.70E-07	**4.47/**9.70E-08	
**Csf3**//colony stimulating factor 3 (granulocyte)	10738051	**7.04/**2.47E-06	**4.34/**7.97E-06	
**Arrdc4**//arrestin domain containing 4	10722720	**2.39/**2.99E-05	**4.32/**9.50E-07	
*Ddit3//DNA-damage inducible transcript 3*	*10895861*	***2.15/*** *1.08E-07*	***4.26/*** *1.52E-09*	
*Junb//jun B proto-oncogene*	*10806585*	***4.03/*** *2.54E-07*	***4.24/*** *8.44E-08*	
**Tiparp**//TCDD-inducible poly(ADP-ribose) polymerase	10815763	**2.64/**1.51E-06	**4.20/**7.06E-08	
**Hbegf**//heparin-binding EGF-like growth factor	10803947	**4.44/**1.55E-05	**4.10/**1.63E-05	
**Cxcl1**//chemokine (C-X-C motif) ligand 1	10775900	**2.97/**3.28E-07	**4.02/**3.72E-08	**2/03/5.02 (HUVEC)**
**Ttc30b**//tetratricopeptide repeat domain 30B	10846293	**−4.13/**3.19E-05	**−1.44/**4.97E-02	−1.38/−1.54 **(HUVEC)**
***Gemin4*** *//gem (nuclear organelle) associated protein*	*10745022*	***−4.08/*** *2.21E-06*	***−2.10/*** *6.59E-05*	
***RGD1566325*** *//similar to regulator of sex-limitation candidate 16*	*10796900*	***−4.15/*** *4.85E-06*	***−2.16/*** *1.38E-04*	
**est** (ncrna: snoRNA)	10765034	**−1.99/**1.58E-06	**−5.01/**4.91E-09	
**est** (ncrna: snoRNA)	10713606	**−3.21/**2.51E-06	**−5.56/**1.42E-07	
**est** (ncrna: snoRNA)	10713604	**−3.37/**7.17E-06	**−7.34/**2.44E-07	

Genes whose differential expression is limited to RVSMC are shown in **bold**. Genes whose expression were also detected in HeLa or HUVEC are shown in **underlined bold**. Genes whose expression was not affected by Na^+^,K^+^-ATPase inhibition in Ca^2+^-depleted cells are shown in *italics*.

### Effects of Ca^2+^-free medium and intracellular Ca^2+^ chelation on gene expression

**Figure 7:**
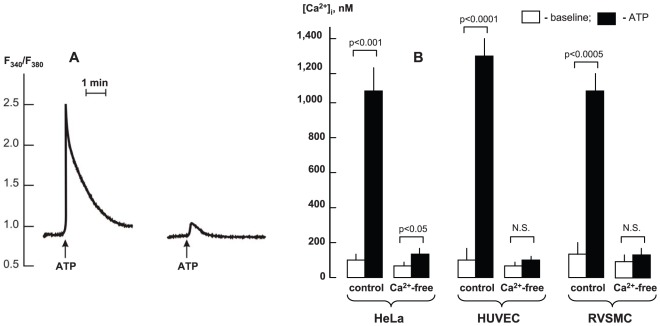
Effect of EGTA and BAPTA on Ca2+ signalling triggered by activation of purinergic receptors. **A.** Representative records showing kinetics of elevation of intracellular Ca^2+^-concentration (F_340_/F_360_ ratio) in HeLa cells triggered by addition of 100 µM ATP. **1** – Control (Ca^2+^-containing medium); **2** – cells were preincubated for 10 min in Ca^2+^-free medium containing 50 µM EGTA and 10 µM BAPTA-AM. **B.** Baseline and maximal values of [Ca^2+^]_i_ in ATP-treated HeLa, HUVEC and RVSMC in control and Ca^2+^-free medium containing 50 µM EGTA and 10 µM BAPTA-AM. Mean ± S.E. values obtained in 4 experiments are shown.

To trigger Ca^2+^-depletion, we incubated cells in Ca^2+^-free medium containing extra- and intracellular Ca^2+^ chelators (50 µM EGTA and 10 µM BAPTA-AM, respectively). Figure 7A displays that in HeLa cells this procedure almost completely abolished sharp elevation of [Ca^2+^]_i_ evoked by activation of purinergic receptors by ATP. We did not reveal any significant elevation of [Ca^2+^]_i_ in Ca^2+^-depleted, ATP-treated HUVEC and RVSMC (Fig. 7B).

**Figure 8:**
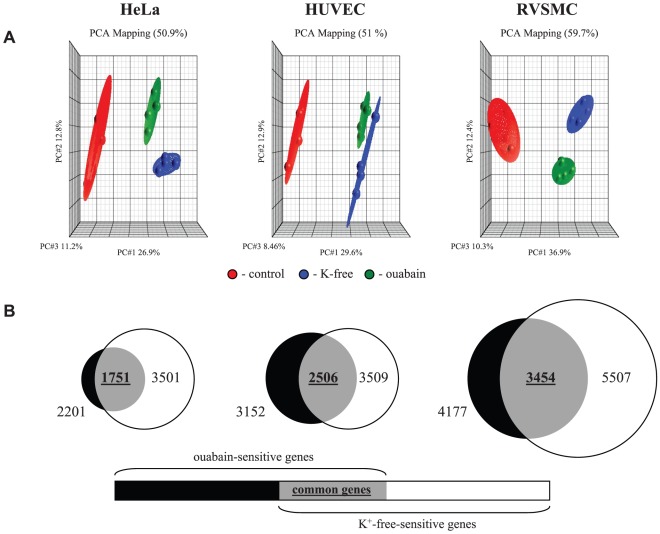
Comparative analysis of the actions of Na^+^,K^+^-ATPase inhibition by ouabain and K^+^-free medium on the transcriptome of Ca^2+^-depleted HeLa, HUVEC and RVSMC. **A.** Principal component analysis of HeLa, HUVEC and RVSMC transcriptomes. Cells were incubated for 3 hr in Ca^2+^-free medium containing 50 µM EGTA and 10 µM BAPTA-AM and processed for oligonucleotide microarray analysis as indicated in the Methods section. Ouabain was added at a final concentration of 3 µM (HeLa and HUVEC) or 3 mM (RVSMC). Ellipsoids highlight portioning of samples based on the type of cell treatment. The principal components in 3-dimensional graphs (PC#1, PC#2 and PC#3) represent the variability in gene expression level within datasets. The total percentage of PCA mapping variability is shown on top. **B.** Total numbers of genes whose expression is altered by ouabain and K^+^-free medium by more than 1.2-fold with p<0.05 are indicated; numbers of genes affected by both stimuli appears in **bold**.

**Figure 9:**
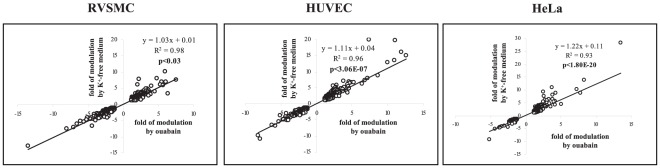
Correlation analysis of transcripts whose expression is altered by ouabain and K^+^-free medium in Ca^2+^-depleted HeLa, HUVEC and RVSMC more than by 1.2-fold with p<0.05. Cells were incubated during 3 hr in Ca^2+^-free medium containing 50 µM EGTA and 10 µM BAPTA-AM. Ouabain was added at a final concentration of 3 µM (HeLa and HUVEC) or 3 mM (RVSMC). The total number of transcripts subjected to analysis is shown in Figure 8B. Transcript expression in control cells was taken as 1.00.

**Figure 10:**
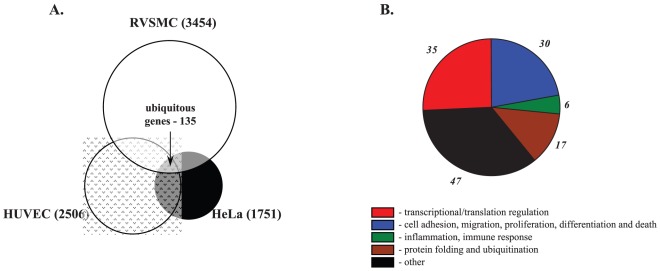
Na^+^_i_,K^+^_i_-sensitive transcriptomes identified in Ca^2+^-depleted cells. **A.** Pie-chart showing the numbers of and cell type-specific, Na^+^_i_,K^+^_i_-sensitive genes detected in HeLa, HUVEC and RVSMC and ubiquitous Na^+^_i_,K^+^_i_-sensitive genes found in all 3 types of cells. Experiments were performed in Ca^2+^-free medium containing 50 µM EGTA and 10 µM BAPTA-AM. **B.** The distribution of ubiquitous and cell type-specific Na^+^_i_,K^+^_i_-sensitive among major functional groups. Digitals shown in ***italics*** correspond to gene numbers in each functional group.

Similarly to the results obtained in Ca^2+^-containing media, PCA found that treatments with ouabain and K^+^-free medium produced dramatic changes in gene expression in Ca^2+^-depleted HUVEC, RVSMC and HeLa cells (Fig. 8A). Figures 8B and Table 7 show that in all types of Ca^2+^-depleted cells, the numbers of differentially expressed genes were increased in comparison to cells incubated in Ca^2+^-containing media (Fig. 3B, Table 2). As in Ca^2+^-containing media, we ascertained significant positive correlations between the differential expression of transcripts triggered by ouabain and K^+^-free medium in Ca^2+^-depleted RVSMC (R^2^ = 0.98; p<0.03), HUVEC (R^2^ = 0.96; p<3×10^−7^) and HeLa cells (R^2^ = 0.93; p<2×10^−20^) (Fig. 9). Importantly, the number of Na^+^_i_,K^+^_i_-sensitive transcripts, i.e. transcripts whose expression is affected by both ouabain and K^+^-free medium, was also increased in the presence of extra- and intracellular Ca^2+^ chelators by ∼3 fold in HeLa and HUVEC and by 2-fold in RVSMC (Fig. 10A, compare to Fig. 6A).

**Table 7:** Total numbers of differentially expressed transcripts in HeLa, HUVEC and RVSMC in 3-hr of Na^+^,K^+^-ATPase inhibition in Ca^2+^-free medium containing extra- and intracellular Ca^2+^ chelators.

	Ouabain-treated cells	Cells treated with K^+^-free medium	Transcripts affected by both stimuli
**HeLa**			
***Up-regulated transcripts***			
Number of transcripts	1007	1633	755
Maximal fold of activation	13.52	28.33	N.A.
***Down-regulated transcripts***			
Number of transcripts*	1204	1868	996
Maximal fold of inhibition	5.18	9.31	N.A.
**HUVEC**			
***Up-regulated transcripts***			
Number of transcripts	1521	1696	1177
Maximal fold of activation	12.58	19.96	N.A.
***Down-regulated transcripts***			
Number of transcripts*	1631	1813	1329
Maximal fold of inhibition	8.19	10.91	N.A.
**RVSMC**			
***Up-regulated transcripts***			
Number of transcripts	2342	3107	1911
Maximal fold of activation	7.60	10.17	N.A.
***Down-regulated transcripts***			
Number of transcripts*	1835	2400	1543
Maximal fold of inhibition	13.49	12.91	N.A.

Transcripts whose expression was altered by more than 1.2-fold with p<0.05 were subjected to analysis. EGTA and BAPTA-AM were added at concentrations of 50 and 10 µM, respectively. Ouabain was added at final concentration of 3 µM (HeLa and HUVEC) or 3 mM (RVSMC). N.A. – non-applicable.

**Figure 11:**
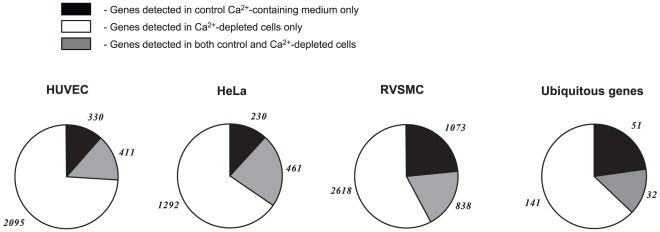
Pie-chart showing the action of Ca^2+^ depletion on Na^+^_i_,K^+^_i_-sensitive transcriptomes. Ca^2+^-depletion was triggered by omission of extracellular Ca^2+^ and addition of 50 µM EGTA and 10 µM BAPTA-AM. The numbers of ubiquitous Na^+^_i_,K^+^_i_-sensitive genes and Na^+^_i_,K^+^_i_-sensitive genes detected in HUVEC, HeLa and RVCSM are shown in ***italics***.

Further analysis disclosed that (***i***) less than 25% of ubiquitous Na^+^_i_,K^+^_i_-sensitive genes were detected in the Ca^2+^-containing media only, (***ii***) the expression of ∼15% of ubiquitous Na^+^_i_,K^+^_i_-sensitive genes was independent on the presence of extra- and intracellular Ca^2+^ chelators, and (***iii***) more than 60% of ubiquitous and cell type-specific Na^+^_i_,K^+^_i_-sensitive genes were found exclusively in Ca^2+^-depleted cells (Fig. 11). In HeLa cells, among the Na^+^_i_,K^+^_i_-sensitive genes whose expression was changed by ouabain or K^+^-free medium by more than 4-fold, we did not observe any genes whose differential expression was abolished by Ca^2+^ depletion (Table 4). In HUVEC and RVSMC this procedure eliminated differential expression of ∼3 and 20% of transcripts, respectively (Tables 5,6).

To further verify the efficacy of Ca^2+^ depletion procedure employed in our study, we compared intracellular concentration of BAPTA, Ca^2+^_i_ and expression of 3 selected genes in HeLa cells incubated in the presence of 10 or 100 µM BAPTA-AM. In control Ca^2+^-containing medium, 3 hr incubation of HeLa cells with ouabain elevated [Ca^2+^]_i_ by ∼35% and increased content of EGR1, PTGS2 and PPP1R15A RNAs by ∼56-, 6- and 9-fold, respectively (Table 8). Addition of 10 µM BAPTA-AM in Ca^2+^-free medium containing 50 µM EGTA completely abolished an increment of [Ca^2+^]_i_ triggered by ouabain and increased the content of EGR1, PTGS2 and PPP1R15A RNAs by ∼6-, 2- and 1.3-fold, respectively. Elevation of BATPA-AM concentration in the incubation medium up to 100 µM increased intracellular [BAPTA] from 127 to 735 µM and augmented expression of EGR1, PTGS2 and PPP1R15A by ∼9-, 2,5- and 2.2-fold, respectively. This action of Ca^2+^-depletion procedure is probably caused by elevation of the passive permeability of the plasma membrane for Na^+^ and [Na^+^]_i_/[K^+^]_i_ ratio detected in EGTA- and BAPTA-AM-treated RVSMC [20]. Importantly, elevation of intracellular [BAPTA] up to 735 µM did not abolished increments of gene expression evoked by 3 hr incubation with ouabain (Table 8).

**Table 8:** Intracellular concentration of BAPTA, Ca^2+^ and expression of EGR1, PTGS2 and PPP1R15A in HeLa cells.

Incubation medium	Ouabain, µM	Intracellular [BAPTA], µM	[Ca^2+^]_i_, nM	Gene expression, arbitrary units,		
				*EGR1*	*PTGS2*	*PPP1R15A*
**1.** Control	0	ND	121±12	1.0	1.0	1.0
	3	ND	164±11*	56.4±2.3***	6.1±0.8***	9.3±1.1***
**2.** Ca^2+^-free+10 µM	0	127±33	97±8	6.0±0.6	1.9±0.3	1.3±0.2
BAPTA-AM	3	ND	92±14	59.9±1.7***	4.8±0.7**	5.1±0.7**
**3.** Ca^2+^-free+100 µM	0	735±101	91±15	9.3±0.8	2.5±0.3	2.2±0.4
BAPTA-AM	3	ND	104±9	55.2±3.4***	7.2±0.8**	6.4±0.5***

HeLa cells were incubated during 3 hrs with or without ouabain in control medium containing 1.8 mM CaCl_2_ (**1**) or in Ca^2+^-free medium containing 50 µM EGTA and 10 or 100 µM BAPTA-AM (**2** and **3**, respectively). Changes in the expression of of EGR1, PTGS2 and PPP1R15A were measured by qRT-PCR. For more details, see Methods section. Means ± S.E. obtained in experiments performed in quadruplicate are shown. ND – these values were not determined.

*, ** and *** - p<0.05, 0.01 and 0.001 compared to values obtained in the absence of ouabain, respectively.

Table 9 lists the ubiquitous Na^+^_i_,K^+^_i_-sensitive genes, which were detected in Ca^2+^-depleted RVSMC, HUVEC and HeLa cells. Similarly to Ca^2+^-containing conditions, ubiquitous Na^+^_i_,K^+^_i_-sensitive transcriptomes in the Ca^2+^-depleted cells were abundant with regulators of transcription/translation, cell cycle control and inflammatory/immune responses (Fig. 10B). Importantly, the actions of elevated [Na^+^]_i_/[K^+^]_i_ ratio on the differential expression of several genes shown in Table 8 in *italics*, such as nuclear receptor *Nr4a1*, anti-proliferative gene *Btg2*, angiogenic inducer *Cyr61*, adrenomedulin *Adm* and regulator of G-protein signaling *Rgs2*, were abolished in the presence of Ca^2+^. We also noted that in Ca^2+^-depleted cells 23 genes or 13% of ubiquitous Na^+^_i_,K^+^_i_-sensitive transcriptome were involved in protein folding and ubiquitination. This is in contrast to 2 genes in the same functional category that were detected in the presence of Ca^2+^ (Fig. 6B). These results are consistent with numerous observations that Ca^2+^-depletion causes endoplasmic reticulum stress and activates unfolded protein response (for review, see [21]).

**Table 9:** Genes whose expression was changed in HeLa, HUVEC and RVSMC by more than 1.2-fold (p≤0.05) in 3 hr of Na^+^,K^+^-ATPase inhibition in Ca^2+^-free medium containing extra- and intracellular Ca^2+^ chelators.

No.	Gene symbol, title	WKY-7		HUVEC		HeLA	
		Fold of activation by		Fold of activation by		Fold of activation by	
		ouabain	K^+^-free medium	Ouabain	K^+^-free medium	ouabain	K^+^-free medium
	***Up-regulated genes***						
**1_t_**	**Fos**//FBJ osteosarcoma oncogene	**6.11**±0.12	**6.97**±0.10	**11.05**±0.14	**19.68**±0.13	**13.52**±0,10	**28.33**±0,08
**2_t_**	**Zfp36**//zinc finger protein 36	**2.77**±0.06	**3.42**±0.06	**12.58**±0.06	**15.10**±0.09	**8.34**±0,11	**13.83**±0,10
**3_t_**	**Fosb**//FBJ osteosarcoma oncogene B	**4.07**±0.11	**5.60**±0.11	**2.70**±0.11	**4.30**±0.07	**3.73**±0,12	**11.09**±0,06
**4_t_**	***Nr4a1*** *//nuclear receptor subfamily 4, group A, member 1*	**1.36**±0.06	**1.83**±0.07	**1.31**±0.03	**1.77**±0.04	**4.23**±0,09	**8.75**±0,09
**5_t_**	**Jun**//Jun oncogene	**1.75**±0.04	**2.16**±0.03	**3.46**±0.06	**4.76**±0.06	**3.81**±0,10	**8.18**±0,08
**6_d_**	**Dusp8**//dual specificity phosphatase 8	**2.34**±0.05	**2.07**±0.05	**3.59**±0.11	**4,73**±0.07	**3.26**±0,10	**6.31**±0,12
**7_d_**	***Btg2*** *//B-cell translocation gene 2, anti-proliferative*	**3.68**±0.07	**4.44**±0.08	**1.42**±0.11	**1.59**±0.11	**2.51**±0,11	**5.36**±0,06
**8_d_**	***Cyr61*** *//cysteine-rich, angiogenic inducer, 61*	**1.58**±0.04	**1.61**±0.04	**2.16**±0.05	**2.43**±0.05	**3.04**±0,09	**4.94**±0,09
**9_d_**	**Ppp1r15a**//protein phosphatase 1, regulatory (inhibitor) subunit 1	**1.33**±0.08	**1.59**±0.07	**1.48**±0.07	**1.63**±0.07	**2.59**±0,11	**3.92**±0,10
**10_d_**	***Nuak2*** *//NUAK family, SNF1-like kinase, 2*	**1.44**±0.09	**1.95**±0.07	**2.91**±0.12	**4.18**±0.14	**1.78**±0,10	**3.85**±0,09
**11_t_**	**Hes1**//hairy and enhancer of split 1 (Drosophila)	**3.56**±0.05	**4.07**±0.05	**2.69**±0.02	**2.96**±0.02	**2.75**±0,04	**3.77**±0,05
**12_i_**	***Adamts1*** *//ADAM metallopeptidase with thrombospondin type 1 motif*	**2.06**±0.07	**2.77**±0.07	**1.60**±0.07	**2.54**±0.09	**2.62**±0,07	**3.56**±0,08
**13_d_**	***Rhob*** *//ras homolog gene family, member B*	**1.72**±0.09	**1.74**±0.10	**1.33**±0.03	**1.52**±0.04	**2.10**±0,08	**3.31**±0,07
**14_t_**	***Id2*** *//inhibitor of DNA binding 2*	**1.29**±0.08	**1.31**±0.07	**1.93**±0.08	**2.71**±0.11	**1.77**±0,09	**3.29**±0,06
**15_o_**	***Adm*** *//adrenomedullin*	**3.28**±0.10	**4.25**±0.09	**3.07**±0.05	**3.76**±0.07	**2.01**±0,09	**3.21**±0,06
**16_d_**	***Dusp1*** *//dual specificity phosphatase 1*	**5.45**±0.10	**6.90**±0.10	**2.71**±0.06	**3.29**±0.06	**2.34**±0,05	**3.13**±0,04
**17_i_**	**Nfkbia**//nuclear factor of kappa light polypeptide gene	**2.53**±0.03	**3.25**±0.04	**1.77**±0.04	**2.18**±0.09	**1.83**±0,08	**3.07**±0,08
**18_t_**	***Ier2*** *//immediate early response 2*	**1.30**±0.02	**1.48**±0.06	**1.82**±0.05	**2.05**±0.06	**2.03**±0,07	**2.86**±0,06
**19_i_**	**Nfkbiz**//nuclear factor of kappa light polypeptide gene	**1.29**±0.06	**1.51**±0.07	**2.44**±0.05	**3.19**±0.05	**2.18**±0,04	**2.64**±0,04
**20_d_**	***Dusp10*** *//dual specificity phosphatase 10*	**1.58**±0.04	**2.08**±0.03	**1.56**±0.06	**2.14**±0.04	**1.99**±0,09	**2.59**±0,06
**21_o_**	***Tiparp*** *//TCDD-inducible poly(ADP-ribose) polymerase*	**1.92**±0.04	**2.01**±0.04	**2.01**±0.04	**2.31**±0.04	**1.89**±0,04	**2.57**±0,04
**22_d_**	**Plk3**//polo-like kinase 3 (Drosophila)	**1.48**±0.04	**1.51**±0.04	**1.52**±0.06	**1.79**±0.09	**1.79**±0,06	**2.52**±0,06
**23_o_**	**Il6**//interleukin 6	**1.84**±0.11	**2.00**±0.11	**1.42**±0.03	**1.27**±0.07	**1.39**±0,13	**2.45**±0,14
**24_t_**	**Maff**//v-maf musculoaponeurotic fibrosarcoma oncogene homolog F	**2.45**±0.12	**2.64**±0.11	**2.21**±0.05	**2.27**±0.05	**1.78**±0,07	**2.24**±0,08
**25_o_**	**Ptgs2**//prostaglandin-endoperoxide synthase 2	**1.78**±0.04	**2.11**±0.03	**1.83**±0.06	**2.12**±0.06	**1.82**±0,09	**2.23**±0,06
**26_t_**	***Jund*** *//Jun D proto-oncogene*	**1.38**±0.04	**1.52**±0.04	**1.73**±0.05	**1.96**±0.07	**1.47**±0,10	**2.16**±0,11
**27_t_**	***Rgs2*** *//regulator of G-protein signaling 2*	**1.35**±0.07	**1.72**±0.08	**2.31**±0.07	**2.99**±0.07	**1.33**±0,08	**2.14**±0,09
**28_d_**	**Ccnl1**//cyclin L1	**1.76**±0.04	**1.72**±0.04	**1.89**±0.06	**2.11**±0.04	**1.52**±0,05	**2.10**±0,03
**29_o_**	***Dusp16*** *//dual specificity phosphatase 16*	**1.38**±0.04	**1.28**±0.03	**2.34**±0.07	**3.43**±0.09	**1.43**±0,07	**2.02**±0,11
**30_d_**	***Lats2*** *//large tumor suppressor 2*	**1.46**±0.06	**1.39**±0.06	**1.43**±0.03	**1.51**±0.03	**1.55**±0,06	**1.94**±0,07
**31_o_**	**Insig1**//insulin induced gene 1	**1.31**±0.03	**1.42**±0.03	**1.81**±0.03	**2.06**±0.03	**1.50**±0,03	**1.89**±0,03
**32_d_**	***Gadd45b*** *//growth arrest and DNA-damage-inducible, beta*	**1.44**±0.08	**1.71**±0.06	**1.68**±0.06	**1.72**±0.08	**1.23**±0,09	**1.87**±0,07
**33_d_**	**Abl2**//v-abl Abelson murine leukemia viral oncogene homolog 2	**2.21**±0.06	**1.86**±0.06	**1.38**±0.03	**1.52**±0.03	**1.51**±0,07	**1.84**±0,09
**34_o_**	***Cd68*** *//Cd68 molecule*	**2.52**±0.17	**1.52**±0.16	**1.31**±0.09	**1.22**±0.10	**1.38**±0,07	**1.83**±0,05
**35_d_**	***Skil*** *//SKI-like oncogene*	**1.44**±0.04	**1.45**±0.05	**1.36**±0.05	**1.52**±0.05	**1.40**±0,07	**1.78**±0,05
**36_t_**	***Crem*** *//cAMP responsive element modulator*	**1.32**±0.06	**1.65**±0.05	**1.51**±0.03	**1.64**±0.05	**1.45**±0,04	**1.68**±0,05
**37_d_**	***Wee1*** *//wee 1 homolog (S. pombe)*	**1.48**±0.06	**1.30**±0.07	**1.52**±0.04	**1.68**±0.05	**1.48**±0,03	**1.63**±0,04
**38_f_**	***Yod1*** *//YOD1 OTU deubiquinating enzyme 1 homolog (S. cerevisiae)*	**1.32**±0.06	**1.31**±0.04	**1.37**±0.04	**1.22**±0.04	**1.55**±0,07	**1.63**±0,07
**39_d_**	***Cd274*** *//CD274 molecule*	**1.31**±0.06	**1.30**±0.06	**4.60**±0.10	**6.81**±0.10	**1.54**±0,05	**1.61**±0,09
**40_d_**	***Hspg2*** *//heparan sulfate proteoglycan 2*	**1.87**±0.03	**1.99**±0.04	**1.36**±0.04	**1.35**±0.04	**1.48**±0,05	**1.58**±0,05
**41_o_**	***Slc19a2*** *//solute carrier family 19 (thiamine transporter)*	**1.29**±0.02	**1.38**±0.06	**1.60**±0.06	**1.56**±0.06	**1.62**±0,07	**1.55**±0,07
**42_o_**	***Zswim6*** *//zinc finger, SWIM-type containing 6*	**1.26**±0.05	**1.33**±0.05	**1.47**±0.02	**1.56**±0.03	**1.37**±0,02	**1.49**±0,02
**43_o_**	***Agrn*** *//agrin*	**1.89**±0.04	**1.92**±0.03	**1.73**±0.06	**1.63**±0.08	**1.34**±0,05	**1.49**±0,05
**44_o_**	***Neu1*** *//sialidase 1 (lysosomal sialidase)*	**1.28**±0.07	**1.49**±0.07	**1.29**±0.09	**1.26**±0.12	**1.38**±0,07	**1.48**±0,05
**45_d_**	***Ptprj*** *//protein tyrosine phosphatase, receptor type*	**1.79**±0.05	**1.81**±0.06	**1.53**±0.06	**1.47**±0.06	**1.39**±0,03	**1.47**±0,03
**46_o_**	***Pfkfb4*** *//6-phosphofructo-2-kinase/fructose-2,6-biphosphatase 4*	**1.23**±0.04	**1.26**±0.04	**1.29**±0.07	**1.30**±0.07	**1.27**±0,08	**1.46**±0,05
**47_o_**	***Lama5*** *//laminin, alpha 5*	**1.37**±0.03	**1.45**±0.03	**1.55**±0.04	**1.55**±0.06	**1.24**±0,04	**1.46**±0,06
**48_d_**	***Cdc26*** *//cell division cycle 26*	**2.10**±0.09	**2.12**±0.08	**1.56**±0.05	**1.62**±0.09	**1.47**±0,05	**1.46**±0,06
**49_t_**	***Cbx6*** *//chromobox homolog 6*	**1.60**±0.03	**1.74**±0.04	**1.59**±0.04	**1.49**±0.04	**1.38**±0,03	**1.42**±0,04
**50_d_**	***Cnnm4*** *//cyclin M4*	**1.27**±0.08	**1.37**±0.06	**1.31**±0.03	**1.37**±0.07	**1.21**±0,09	**1.42**±0,09
**51_o_**	**Txnip**//thioredoxin interacting protein	**3.81**±0.07	**3.72**±0.07	**3.23**±0.05	**2.40**±0.08	**1.31**±0,06	**1.40**±0,04
**52_f_**	***Skp1*** *//S-phase kinase-associated protein 1*	**1.30**±0.04	**1.30**±0.03	**1.41**±0.08	**1.52**±0.09	**1.30**±0,05	**1.38**±0,05
**53_d_**	***Ltbp2*** *//latent transforming growth factor beta binding protein 2*	**1.27**±0.03	**1.32**±0.04	**1.24**±0.04	**1.21**±0.04	**1.27**±0,04	**1.37**±0,04
**54_o_**	***B4galt5*** *//UDP-Gal:betaGlcNAc beta 1,4-galactosyltransferase*	**1.60**±0.03	**1.79**±0.03	**1.28**±0.02	**1.20**±0.02	**1.42**±0,02	**1.36**±0,03
**55_i_**	***Relt*** *//RELT tumor necrosis factor receptor*	**1.51**±0.05	**1.31**±0.05	**1.33**±0.05	**1.25**±0.07	**1.39**±0,03	**1.36**±0,07
**56_t_**	***Rfx1*** *//regulatory factor X, 1 (influences HLA class II expression)*	**1.29**±0.07	**1.23**±0.07	**1.36**±0.11	**1.27**±0.10	**1.31**±0,04	**1.34**±0,08
**57_o_**	***Pip5k1c*** *//phosphatidylinositol-4-phosphate 5-kinase, type I*	**1.26**±0.05	**1.29**±0.05	**1.25**±0.06	**1.23**±0.06	**1.23**±0,02	**1.33**±0,06
**58_d_**	***Efhd2*** *//EF-hand domain family, member D2*	**1.23**±0.04	**1.36**±0.04	**1.53**±0.09	**1.52**±0.11	**1.23**±0,04	**1.32**±0,07
**59_o_**	***Slc16a3*** *//solute carrier family 16, member 3*	**2.17**±0.11	**2.30**±0.11	**1.36**±0.07	**1.30**±0.07	**1.26**±0,04	**1.32**±0,06
**60_d_**	***Ccnd1*** *//cyclin D1*	**1.25**±0.03	**1.33**±0.03	**1.42**±0.04	**1.35**±0.03	**1.25**±0,03	**1.32**±0,04
**61_o_**	***Gla*** *//galactosidase, alpha*	**1.72**±0.03	**1.61**±0.06	**1.34**±0.09	**1.50**±0.07	**1.31**±0,06	**1.30**±0,08
**62_o_**	***Abca2*** *//ATP-binding cassette, sub-family A (ABC1), member 2*	**1.48**±0.04	**1.45**±0.05	**1.37**±0.05	**1.28**±0.07	**1.22**±0,04	**1.27**±0,04
**63_o_**	***Epha4*** *//Eph receptor A4*	**2.03**±0.08	**2.28**±0.09	**1.67**±0.06	**1.83**±0.06	**1.28**±0,06	**1.27**±0,08
**64_t_**	**Nfya**//nuclear transcription factor-Y alpha	**1.40**±0.06	**1.27**±0.05	**1.35**±0.02	**1.30**±0.07	**1.47**±0,06	**1.26**±0,05
**65_o_**	***Megf9*** *//multiple EGF-like-domains 9*	**1.40**±0.05	**1.55**±0.05	**1.27**±0.07	**1.29**±0.07	**1.26**±0,04	**1.26**±0,08
**66_t_**	***Arid3b*** *//AT rich interactive domain 3B (Bright like)*	**1.46**±0.05	**1.24**±0.05	**1.57**±0.07	**1.51**±0.07	**1.29**±0,11	**1.26**±0,10
**67_o_**	***Pnkd*** *//paroxysmal nonkinesiogenic dyskinesia*	**1.55**±0.08	**1.71**±0.08	**1.42**±0.05	**1.27**±0.07	**1.28**±0,04	**1.26**±0,05
**68_t_**	***Sart3*** *//squamous cell carcinoma antigen recognized by T-cells 3*	**1.34**±0.05	**1.28**±0.05	**1.53**±0.04	**1.49**±0.04	**1.23**±0,08	**1.24**±0,05
**69_o_**	***Myl6*** *//myosin, light chain 6, alkali, smooth muscle and non-muscle*	**1.22**±0.01	**1.24**±0.01	**1.22**±0.04	**1.22**±0.04	**1.30**±0,04	**1.23**±0,04
**70_o_**	***Slc2a12*** *//solute carrier family 2 (facilitated glucose transport)*	**1.45**±0.07	**1.58**±0.06	**1.34**±0.09	**1.45**±0.10	**1.34**±0,08	**1.22**±0,05
	***Down-regulated genes***						
**1_d_**	***Chac1*** *//ChaC, cation transport regulator homolog 1 (E. coli)*	**−1.80**±0.07	**−2.21**±0.10	**−4.07**±0.12	**−5.17**±0.20	**−5.18**±0,09	**−9.31**±0,11
**2_f_**	***Herpud1*** *//homocysteine-inducible, endoplasmic reticulum stress-ind*	**−2.66**±0.06	**−3.03**±0.05	**−2.30**±0.06	**−3.12**±0.05	**−1.95**±0,07	**−2.64**±0,07
**3_t_**	***Sdf2l1*** *//stromal cell-derived factor 2-like 1*	**−1.82**±0.07	**−1.63**±0.07	**−3.03**±0.04	**−3.67**±0.03	**−1.80**±0,08	**−2.36**±0,05
**4_t_**	**Hoxb5**//homeo box B5	**−1.57**±0.11	**−1.53**±0.11	**−1.96**±0.06	**−2.19**±0.08	**−1.92**±0,06	**−2.33**±0,04
**5_o_**	***Asns*** *//asparagine synthetase*	**−1.34**±0.05	**−1.43**±0.04	**−1.67**±0.06	**−1.67**±0.09	**−1.64**±0,07	**−1.96**±0,04
**6_t_**	***Trnt1*** *//tRNA nucleotidyl transferase, CCA-adding, 1*	**−1.37**±0.14	**−1.69**±0.08	**−1.20**±0.06	**−1.25**±0.07	**−1.51**±0,10	**−1.94**±0,07
**7_f_**	***Dnajc3*** *//DnaJ (Hsp40) homolog, subfamily C, member 3*	**−2.12**±0.06	**−2.17**±0.05	**−2.25**±0.04	**−2.51**±0.03	**−1.72**±0,06	**−1.91**±0,06
**8_t_**	***Cebpg*** *//CCAAT/enhancer binding protein (C/EBP), gamma*	**−1.30**±0.04	**−1.45**±0.03	**−1.81**±0.06	**−1.91**±0.05	**−1.47**±0,07	**−1.91**±0,08
**9_o_**	***Riok2*** *//RIO kinase 2 (yeast)*	**−1.68**±0.05	**−1.60**±0.05	**−1.59**±0.06	**−1.53**±0.06	**−1.61**±0,06	**−1.89**±0,08
**10_o_**	***Ficd*** *//FIC domain containing*	**−4.62**±0.09	**−4.59**±0.08	**−3.75**±0.15	**−3.79**±0.17	**−1.71**±0,09	**−1.87**±0,09
**11_o_**	***Pdia4*** *//protein disulfide isomerase family A, member 4*	**−1.82**±0.05	**−1.70**±0.05	**−1.58**±0.04	**−1.67**±0.04	**−1.64**±0,07	**−1.86**±0,07
**12_o_**	***Pck2*** *//phosphoenolpyruvate carboxykinase 2 (mitochondrial)*	**−1.50**±0.05	**−1.49**±0.05	**−1.95**±0.05	**−2.06**±0.08	**−1.79**±0,05	**−1.86**±0,06
**13_t_**	***Cars*** *//cysteinyl-tRNA synthetase*	**−1.41**±0.04	**−1.97**±0.04	**−1.67**±0.05	**−1.89**±0.06	**−1.51**±0,10	**−1.85**±0,09
**14_d_**	***Dnajb9*** *//DnaJ (Hsp40) homolog, subfamily B, member 9*	**−1.91**±0.05	**−2.09**±0.06	**−4.30**±0.11	**−4.87**±0.07	**−1.73**±0,10	**−1.80**±0,08
**15_o_**	***Stc2*** *//stanniocalcin 2*	**−1.99**±0.03	**−2.03**±0.05	**−3.35**±0.07	**−3.40**±0.09	**−2.17**±0,10	**−1.79**±0,11
**16_o_**	***Zbed3*** *//zinc finger, BED-type containing 3*	**−1.52**±0.11	**−1.33**±0.10	**−1.65**±0.08	**−1.88**±0.07	**−1.56**±0,09	**−1.76**±0,12
**17_o_**	**Rpp40**//ribonuclease P 40 subunit (human)	**−1.35**±0.08	**−1.40**±0.09	**−1.46**±0.04	**−1.24**±0.05	**−1.31**±0,07	**−1.74**±0,07
**18_t_**	***Trim27*** *//tripartite motif-containing 27*	**−1.87**±0.07	**−1.84**±0.06	**−1.45**±0.03	**−1.30**±0.03	**−1.59**±0,05	**−1.72**±0,07
**19_f_**	***Hyou1*** *//hypoxia up-regulated 1*	**−2.24**±0.08	**−2.25**±0.08	**−1.71**±0.05	**−1.87**±0.05	**−1.42**±0,04	**−1.71**±0,04
**20_t_**	**Mrpl46**//mitochondrial ribosomal protein L46	**−1.34**±0.05	**−1.35**±0.06	**−1.36**±0.04	**−1.60**±0.05	**−1.33**±0,04	**−1.70**±0,09
**21_t_**	***Xbp1*** *//X-box binding protein 1*	**−1.67**±0.04	**−1.86**±0.03	**−1.46**±0.07	**−1.61**±0.07	**−1.30**±0,05	**−1.70**±0,04
**22_f_**	***Sel1l*** *//sel-1 suppressor of lin-12-like (C. elegans)*	**−1.58**±0.07	**−1.74**±0.07	**−2.20**±0.01	**−2.37**±0.02	**−1.57**±0,06	**−1.68**±0,07
**23_o_**	***Ankrd49*** *//ankyrin repeat domain 49*	**−1.43**±0.04	**−1.33**±0.06	**−1.50**±0.11	**−1.49**±0.08	**−1.51**±0,10	**−1.66**±0,08
**24_o_**	***Lin37*** *//lin-37 homolog (C. elegans)*	**−1.40**±0.06	**−1.34**±0.04	**−1.45**±0.06	**−1.62**±0.05	**−1.46**±0,06	**−1.64**±0,07
**25_t_**	***Alkbh8*** *//alkB, alkylation repair homolog 8 (E. coli)*	**−1.92**±0.05	**−1.92**±0.07	**−1.37**±0.07	**−1.26**±0.05	**−1.37**±0,10	**−1.64**±0,08
**26_o_**	***Gpt2*** *//glutamic pyruvate transaminase*	**−1.35**±0.07	**−1.38**±0.08	**−1.64**±0.06	**−1.58**±0.05	**−1.70**±0,08	**−1.63**±0,04
**27_o_**	***Kctd6*** *//potassium channel tetramerisation domain containing 6*	**−1.80**±0.10	**−1.65**±0.09	**−1.51**±0.07	**−1.69**±0.13	**−1.41**±0,08	**−1.62**±0,08
**28_t_**	***Hoxb6*** *//homeo box B6*	**−1.60**±0.08	**−1.58**±0.08	**−1.38**±0.10	**−1.33**±0.08	**−1.38**±0,07	**−1.58**±0,08
**29_d_**	***Ddit4*** *//DNA-damage-inducible transcript 4*	**−2.29**±0.02	**−1.97**±0.05	**−5.09**±0.10	**−3.69**±0.13	**−1.84**±0,05	**−1.58**±0,06
**30_o_**	***Slc25a28*** *//solute carrier family 25, member 28*	**−1.85**±0.05	**−1.83**±0.04	**−1.41**±0.07	**−1.53**±0.07	**−1.28**±0,06	**−1.57**±0,05
**31_o_**	***Cth*** *//cystathionase (cystathionine gamma-lyase)*	**−2.51**±0.05	**−2.62**±0.06	**−2.22**±0.09	**−2.20**±0.12	**−1.89**±0,09	**−1.54**±0,09
**32_f_**	***Rnf217*** *//ring finger protein 217*	**−1.31**±0.09	**−1.33**±0.09	**−2.73**±0.08	**−2.86**±0.05	**−1.28**±0,09	**−1.53**±0,10
**33_f_**	***Rnf146*** *//ring finger protein 146*	**−1.30**±0.02	**−1.21**±0.03	**−1.43**±0.03	**−1.59**±0.06	**−1.35**±0,05	**−1.50**±0,07
**34_f_**	***Fbxo42*** *//F-box protein 42*	**−1.42**±0.05	**−1.41**±0.05	**−1.38**±0.04	**−1.41**±0.05	**−1.43**±0,04	**−1.50**±0,06
**35_o_**	***Creld2*** *//cysteine-rich with EGF-like domains 2*	**−3.27**±0.09	**−2.99**±0.08	**−1.55**±0.05	**−1.51**±0.05	**−1.50**±0,06	**−1.49**±0,07
**36_f_**	***Hspa5*** *//heat shock protein 5*	**−1.28**±0.03	**−1.23**±0.03	**−1.57**±0.03	**−1.72**±0.01	**−1.42**±0,04	**−1.48**±0,07
**37_o_**	***Ttc14*** *//tetratricopeptide repeat domain 14*	**−1.23**±0.08	**−1.41**±0.06	**−1.42**±0.04	**−1.69**±0.06	**−1.27**±0,06	**−1.48**±0,08
**38_d_**	***Trib3*** *//tribbles homolog 3 (Drosophila)*	**−2.60**±0.03	**−3.86**±0.06	**−1.26**±0.05	**−1.41**±0.07	**−1.26**±0,07	**−1.46**±0,07
**39_d_**	***Jag1*** *//jagged 1*	**−1.45**±0.04	**−1.76**±0.02	**−1.36**±0.04	**−1.33**±0.05	**−1.39**±0,04	**−1.45**±0,05
**40_d_**	***Esco1*** *//establishment of cohesion 1 homolog 1 (S. cerevisiae)*	**−1.40**±0.04	**−1.43**±0.04	**−1.36**±0.09	**−1.21**±0.08	**−1.66**±0,12	**−1.45**±0,10
**41_t_**	***Exosc3*** *//exosome component 3*	**−1.29**±0.06	**−1.25**±0.06	**−1.46**±0.06	**−1.33**±0.06	**−1.27**±0,10	**−1.44**±0,12
**42_o_**	***Slc33a1*** *//solute carrier family 33 (acetyl-CoA transporter)*	**−1.88**±0.10	**−2.21**±0.09	**−1.92**±0.08	**−1.89**±0.06	**−1.41**±0,05	**−1.44**±0,06
**43_d_**	***Tbccd1*** *//TBCC domain containing 1*	**−1.35**±0.06	**−1.28**±0.04	**−1.38**±0.04	**−1.40**±0.08	**−1.41**±0,06	**−1.44**±0,03
**44_o_**	***Znf509*** *//zinc finger protein 509*	**−1.39**±0.04	**−1.26**±0.04	**−1.42**±0.12	**−1.29**±0.10	**−1.41**±0,02	**−1.43**±0,07
**45_o_**	***Slc25a37*** *//solute carrier family 25, member 37*	**−2.17**±0.05	**−1.89**±0.07	**−1.25**±0.05	**−1.20**±0.06	**−1.33**±0,06	**−1.42**±0,06
**46_f_**	***Dnajb11*** *//DnaJ (Hsp40) homolog, subfamily B, member 11*	**−1.33**±0.05	**−1.25**±0.03	**−1.62**±0.06	**−1.80**±0.04	**−1.28**±0,05	**−1.42**±0,06
**47_f_**	***Fem1b*** *//feminization 1 homolog b (C. elegans)*	**−1.71**±0.03	**−1.60**±0.03	**−1.37**±0.05	**−1.39**±0.05	**−1.31**±0,04	**−1.39**±0,04
**48_f_**	***Trim23*** *//tripartite motif-containing 23*	**−1.37**±0.05	**−1.28**±0.05	**−1.57**±0.08	**−1.63**±0.09	**−1.40**±0,09	**−1.39**±0,09
**49_t_**	***Snapc3*** *//small nuclear RNA activating complex, polypeptide 3*	**−1.36**±0.05	**−1.40**±0.05	**−1.35**±0.05	**−1.41**±0.04	**−1.24**±0,07	**−1.37**±0,06
**50_t_**	***Gars*** *//glycyl-tRNA synthetase*	**−1.31**±0.02	**−1.33**±0.02	**−1.24**±0.02	**−1.25**±0.03	**−1.25**±0,03	**−1.36**±0,03
**51_o_**	***Cyp2r1*** *//cytochrome P450, family 2, subfamily r, polypeptide 1*	**−1.37**±0.07	**−1.63**±0.08	**−1.31**±0.07	**−1.34**±0.07	**−1.31**±0,09	**−1.36**±0,06
**52_o_**	***Napb*** *//N-ethylmaleimide-sensitive factor attachment prote*	**−1.31**±0.10	**−1.27**±0.11	**−1.32**±0.12	**−1.43**±0.10	**−1.36**±0,07	**−1.35**±0,10
**53_o_**	***Oraov1*** *//oral cancer overexpressed 1*	**−1.29**±0.04	**−1.42**±0.08	**−1.27**±0.09	**−1.30**±0.09	**−1.22**±0,08	**−1.34**±0,07
**54_o_**	***Piga*** *//phosphatidylinositol glycan anchor biosynthesis, class A*	**−2.27**±0.07	**−2.92**±0.06	**−2.29**±0.04	**−2.56**±0.03	**−1.33**±0,06	**−1.34**±0,06
**55_f_**	***Dnajb5*** *//DnaJ (Hsp40) homolog, subfamily B, member 5*	**−1.24**±0.04	**−1.22**±0.05	**−1.76**±0.06	**−1.70**±0.06	**−1.46**±0,09	**−1.33**±0,07
**56_f_**	***Siah2*** *//seven in absentia 2*	**−1.34**±0.02	**−1.29**±0.06	**−1.47**±0.09	**−1.50**±0.12	**−1.27**±0,10	**−1.33**±0,07
**57_t_**	***Ccdc101*** *//coiled-coil domain containing 101*	**−1.22**±0.06	**−1.32**±0.06	**−1.63**±0.10	**−1.52**±0.08	**−1.30**±0,04	**−1.33**±0,08
**58_t_**	***Ccdc76*** *//coiled-coil domain containing 76*	**−1.23**±0.05	**−1.53**±0.06	**−1.26**±0.05	**−1.26**±0.05	**−1.35**±0,06	**−1.33**±0,05
**59_d_**	***Rad17*** *//RAD17 homolog (S. pombe)*	**−1.22**±0.10	**−1.42**±0.05	**−1.30**±0.08	**−1.32**±0.07	**−1.29**±0,09	**−1.32**±0,07
**60_t_**	***Lrrfip1*** *//leucine rich repeat (in FLII) interacting protein 1*	**−1.49**±0.08	**−1.52**±0.07	**−2.92**±0.16	**−3.62**±0.21	**−1.30**±0,09	**−1.32**±0,10
**61_t_**	***Zxdc*** *//ZXD family zinc finger C*	**−1.31**±0.06	**−1.27**±0.06	**−1.39**±0.11	**−1.31**±0.09	**−1.27**±0,11	**−1.28**±0,10
**62_t_**	***Zrsr2*** *//zinc finger (CCCH type), RNA binding motif and serine/a*	**−1.40**±0.07	**−1.68**±0.09	**−1.31**±0.06	**−1.24**±0.07	**−1.23**±0,07	**−1.28**±0,07
**63_f_**	***Txndc11*** *//thioredoxin domain containing 11*	**−1.31**±0.05	**−1.32**±0.04	**−1.26**±0.02	**−1.34**±0.04	**−1.22**±0,03	**−1.24**±0,06
**64_o_**	***Ibtk*** *//inhibitor of Bruton agammaglobulinemia tyrosine kinase*	**−1.37**±0.07	**−1.38**±0.06	**−1.41**±0.03	**−1.37**±0.04	**−1.24**±0,06	**−1.23**±0,07
**65_f_**	***Gopc*** *//golgi associated PDZ and coiled-coil motif containing*	**−1.21**±0.07	**−1.28**±0.06	**−1.40**±0.05	**−1.39**±0.07	**−1.24**±0,06	**−1.23**±0,04

Genes whose expression was not affected by ouabain or K^+^-free medium in the presence of Ca^2+^ in at least one of cell types are shown in ***italics***. Genes whose expression was not affected by ouabain or K^+^-free medium in the presence of Ca^2+^ in all 3 types of cells shown in ***underlined italics***. Gene functions are indicated in the left column as: **t** – regulators of transcription/translation, RNA processing and degradation; **d** – regulators of cell adhesion, migration, proliferation, differentiation and death; **f** - protein folding and ubiquitination; **i** – inflammation and immune response; **o** – other functional categories and genes withy unknown function.

**Figure 12:**
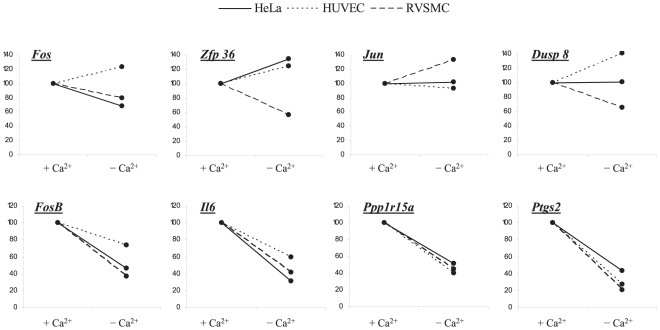
Effect of Ca^2+^-depletion on expression of ubiquitous Na^+^_i_,K^+^_i_-sensitive genes in HeLa, HUVEC and RVSMC. Cells were incubated during 3 hrs in control medium containing 1.8 mM CaCl_2_ or in Ca^2+^-containing K^+^-free medium. To trigger Ca^2+^-depletion, CaCl_2_ was omitted and 50 µM EGTA and 10 µM BAPTA-AM were added. Increments of gene expression triggered by Na^+^,K^+^-ATPase inhibition in Ca^2+^-containing medium were taken as 100%. For absolute values of gene expression, see Tables 3 and 8.

Based on this work we selected 8 ubiquitous Na^+^_i_,K^+^_i_-sensitive genes whose expression was increased by more than 5-fold in Ca^2+^-containing media, and whose augmented expression was preserved in the presence of extra- and intracellular Ca^2+^ chelators. As seen in Figure 12, Ca^2+^-depletion strongly attenuated (but did not completely abolish) Na^+^_i_/K^+^_i_-dependent changes in gene expression for *FosB, Il6, Pppr1r15a* and *Ptgs2* in all cell types. In contrast, Ca^2+^-depletion slightly decreased, did not affect, or increased expression of *Fos, Zfp36, Jun* and *Dusp8* in a cell type-specific manner. These results further illustrate that (***i***) [Ca^2+^]_i_ elevation is not obligatory for changes in gene expression, and (***ii***) Ca^2+^_i_-dependent signaling may have complex effects on ubiquitous and cell type specific Na^+^_i_,K^+^_i_-sensitive transcriptomes.

## Discussion

In the present study we utilized genome-wide Affymetrix arrays to identify [Na^+^]_i_/[K^+^]_i_-sensitive transcriptomes in 3 diverse cell lines. Based on the results presented here we can conclude that elevation of the [Na^+^]_i_/[K^+^]_i_-ratio causes robust changes in gene expression in both ubiquitous and cell type-dependent manners. The number of regulated transcripts was high and represented more than 2% of the total genome. Surprisingly, Ca^2+^-depletion increased rather than decreased number of the [Na^+^]_i_/[K^+^]_i_-sensitive genes. These findings point to the existence of novel, yet unidentified Ca^2+^_i_-independent mechanisms of transcriptional regulation, which are determined by the intracellular concentration of monovalent cations.

### Characterization of ubiquitous Na^+^_i_,K^+^_i_-sensitive transcriptome

We observed that sustained Na^+^,K^+^-ATPase inhibition by ouabain led to the differential expression of 886 transcripts in primary cultured human endothelial cells, 819 transcripts in human epitheloid carcinoma cell line and 3191 transcripts in smooth muscle cells obtained from the rat aorta (Fig. 2). Differentially regulated genes represent ∼3 and ∼10% of genes in human and rat genome, respectively. Keeping in mind that ouabain may affect signaling pathways independently of inhibition of Na^+^,K^+^-ATPase [14], [15], we tested K^+^-free medium as an alternative approach to elevation of the [Na^+^]_i_/[K^+^]_i_ ratio. Three hour incubation of cells in K^+^-free medium resulted in gain of Na^+^_i_ and loss of K^+^_i_ that were quantitatively similar to those triggered by ouabain (Fig. 1). We found highly significant positive correlations between the levels of transcripts impacted by both stimuli (Fig. 4). These data strongly indicate that differential gene expression is evoked by elevation of the [Na^+^]_i_/[K^+^]_i_ ratio rather than by Na^+^_i_,K^+^_i_-independent mechanisms.

Among 684, 737 and 1839 Na^+^_i_,K^+^_i_-sensitive transcripts detected in HeLa, HUVEC and RVSMC, we discerned 80 genes whose expression was increased up to 60-fold or decreased up to ∼3-fold independently of the origin of cultured cells (Table 3). It should be noted that because of the retarded kinetics of elevation of the [Na^+^]_i_/[K^+^]_i_ ratio in human cells (Fig. 3), the number of Na^+^_i_,K^+^_i_-sensitive genes in HeLa and HUVEC as well as the number of ubiquitous Na^+^_i_,K^+^_i_-sensitive genes is probably underestimated.

**Figure 13:**
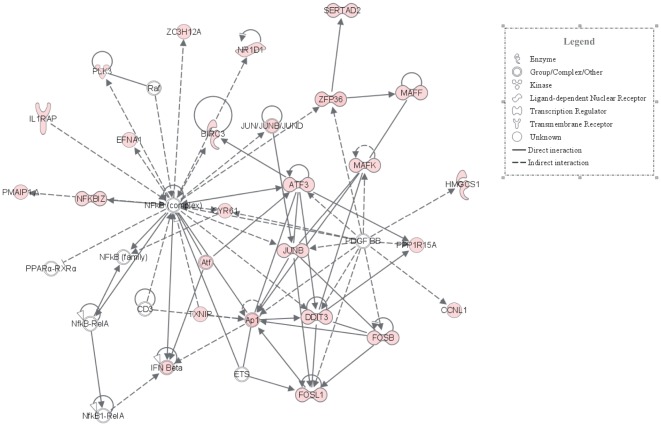
The signaling network possessing the highest score of 48 for association with differential expression of ubiquitous Na^+^_i_,K^+^_i_-sensitive genes. The *p*-value and geometric fold change for each gene listed in Table 3 were overlaid onto a global molecular network developed from information within the IPA Knowledge Base. Genes are represented as nodes of various shapes to represent the functional category of gene product as shown in the top corner, and the biological relationship between two nodes is represented as a line. The up-regulated Na^+^_i_,K^+^_i_-sensitive genes are shown in **pink**. Note that network's Score of 48 is equal to -log(Fisher's Exact test result). It means that there is a 1 in 10^48^ chance of getting a network from Ingenuity Knowledge Base containing at least the same number of eligible molecules by chance when randomly picking molecules derived form 80 ubiquitous Na^+^_i_,K^+^_i_-sensitive genes listed in Table 3.

Functional analysis demonstrated that almost the half of the ubiquitous Na^+^_i_,K^+^_i_-sensitive genes belonged to transcription and translation regulators (Fig. 6). Important examples include *Egr1, Fos, Fosb, Atf3, Jun, Ddit3, Junb, Cyr61*. Although functional characterization is somewhat artificial – because genes are usually multifunctional and fall into several categories – the relative content of transcriptional regulators identified in the ubiquitous Na^+^_i_,K^+^_i_-sensitive transcriptome was ∼7-fold higher than in total human genome [22]. These findings strongly suggest that heightened expression of ubiquitous Na^+^_i_,K^+^_i_-sensitive regulators of transcription underlie ubiquitous and cell-type specific transcriptomic modifications triggered by elevation of the [Na^+^]_i_/[K^+^]_i_. This conclusion is supported by analysis of the top gene expression network revealed using the IPA Knowledge Base software. Indeed, Figure 13 illustrates the central role of ubiquitous Na^+^_i_/K^+^_i_-sensitive regulators of gene expression such as *Fos, FosB, Jun, JunB, Atf3, Cyr61* in the triggering of diverse cellular signals.

**Figure 14:**
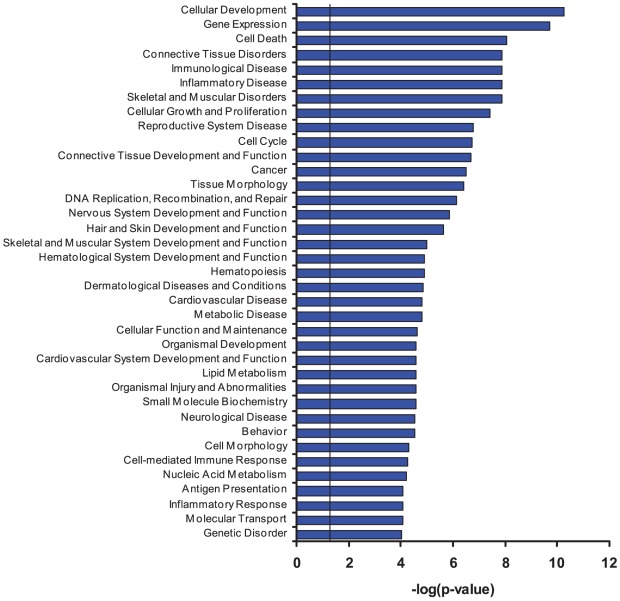
Top significantly altered biological functions associated with differential expression of ubiquitous Na^+^_i_,K^+^_i_-sensitive genes. The *p*-value and geometric fold change for each gene listed in Table 3 were imported into Ingenuity Pathway Analysis. The significance criteria with a threshold of p = 0.05 (or 1.3 when expressed as -log(p-value) is shown by line.

Figure 14 shows that cellular development, gene expression, cell death, immunological and inflammatory responses are among top altered biological functions controlled by ubiquitous Na^+^_i_/K^+^_i_-sensitive transcriptome and mapped in this software with p<10^−5^. Importantly, cellular responses triggered by differential expression of Na^+^_i_,K^+^_i_-sensitive genes might be cell type-specific. Thus, for example 24 hr incubation with ouabain resulted in death of renal epithelial cells [23] but rescued RVSMC from apoptosis triggered by serum deprivation [24].

### Ca^2+^-depletion increases the number of [Na^+^]_i_/[K^+^]_i_-sensitive genes

Numerous studies have demonstrated that elevation of the [Na^+^]_i_/[K^+^]_i_ ratio heightens [Ca^2+^]_i_ via activation of Na^+^_i_/Ca^2+^_o_ exchanger as well as via depolarization and activation of voltage-gated Ca^2+^ channels (for review, see [9], [10]). It has also been well-documented that elevation of [Ca^2+^]_i_ affects gene expression by activation of SRE via Ras-Raf-Erk-Elk1-mediated signaling, CRE via phosphorylation of CRE binding protein and nuclear factor AT (NFAT) binding sites via NFAT dephosphorylation by calcineurin [7], [25]–[27]. Unexpectedly, we found that in the presence of extra- and intracellular Ca^2+^ chelators (EGTA and BAPTA, respectively) the number of cell type-specific and ubiquitous Na^+^_i_,K^+^_i_-sensitive genes was increased rather than decreased. Importantly, differential expression of more than 50% of ubiquitous and cell type-specific Na^+^_i_,K^+^_i_-sensitive genes was detected in Ca^2+^-depleted cells only (Fig. 11). Furthermore, the expression of several genes such as *Fos, Zfp36, Jun* and *Dusp8* was not affected or even activated in the presence of extra- and intracellular Ca^2+^ chelators (Fig. 12).

Recently, Akita and Okada reported that activation of volume-sensitive anion channels in astrocytes by bradykinin was suppressed by addition of BAPTA-AM at concentration higher than 100 µM [28]. Thus, it may be proposed that at concentration 10 µM used in our study BAPTA-AM was unable completely abolish elevation of [Ca^2+^]_i_ triggered by sustained inhibition of the Na^+^/K^+^-ATPase. However, data listed below did not support this assumption. *First*, 30 min incubation in Ca^2+^-free medium containing 10 µM BAPTA-AM and 50 µM EGTA sharply suppressed or completely abolished an increment of [Ca^2+^]_i_ triggered by activation of purinergic receptors (Fig. 7). *Second*, in contrast to overwhelming number of electrically excitable cells, 2 hr exposure of RVSMC to ouabain did not affect [Ca^2+^]_i_ but sharply increased expression of *Fos* and *Jun* superfamily IRG [12]. Here, we demonstrated that addition of 50 µM EGTA and 10 µM BAPTA-AM completely abolished a modest increment of [Ca^2+^]_i_ triggered by 3 hr incubation of HeLa cells with ouabain whereas augmented expression of EGR1, PTGS2 and PPP1R15A was preserved (Table 8). *Third*, in the presence of 10 µM BAPTA-AM, intracellular concentration BAPTA in HeLa cells reached a value of ∼130 µM (Table 8). Based on the volume of intracellular water (∼2 µl/mg of protein), the intracellular BAPTA content on these cells (∼260 pmol/mg protein) was sufficient to bind the total pool of intracellular exchangeable calcium (∼200 pmol/mg protein) [13]. *Fourth*, elevation of intracellular [BAPTA] up to ∼700 µM did not abolished increments of gene expression evoked by ouabain (Table 8). It is important to note that long-term exposure of cells to Ca^2+^ chelators at higher concentrations causes diverse side-effects including elevated permeability of the plasma membrane for Na^+^ [20]. Moreover, because of the high affinity to other di- and trivalent cations [29], Ca^2+^ chelators can affect transcriptome via irreversible conformational transition and inactivation of transcriptional adaptor Zn^2+^-binding domain [30]. Viewed collectively, these data strongly indicate that besides canonical Ca^2+^-mediated signalling, gain of [Na^+^]_i_ and/or loss of [K^+^]_i_ influence gene expression via Ca^2+^_i_-independent mechanism.

### Physiological and pathophysiological implications

**Figure 15:**
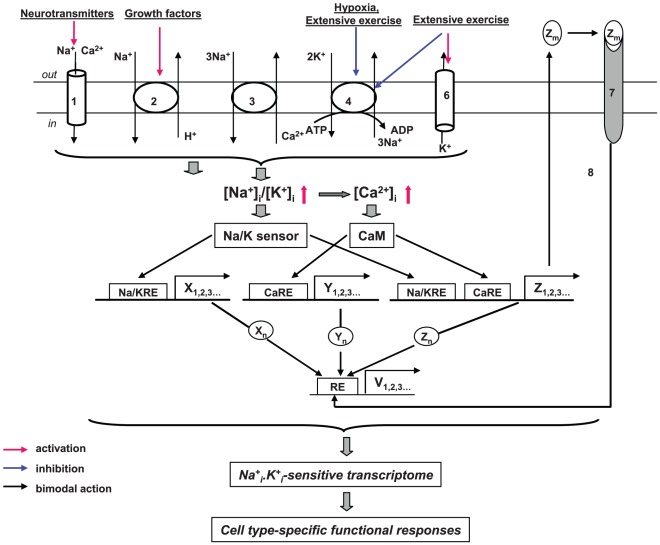
Mechanisms underlying transcriptomic alterations in normal and pathophysiological conditions: *a working hypothesis*. Activation of Na^+^-permeable channels such as NMDA receptors (**1**), Na^+^/H^+^ exchanger (**2**), voltage-gated K^+^ channels (**6**) and inhibition of Na^+^,K^+^-ATPase (**4**) lead to elevation of the [Na^+^]_i_/[K^+^]_i_ ratio. In cells abundant with Na^+^/Ca^2+^ exchanger (**3**), the dissipation of transmembrane gradients of monovalent cations is accompanied by elevation of [Ca^2+^]_i_. Elevation of the [Na^+^]_i_/[K^+^]_i_ ratio affects the expression of **X_1,2,3…_** and **Z_1,2,3…_** genes via activation of unknown Na/K sensor(s) and Na/K response elements (**Na/KRE**). Expression of **Z_1,2,3…_** genes is also subjected to regulation by elevated [Ca^2+^]_i_ via its interaction with calmodulin (**CaM**) and other Ca^2+^_i_ sensors and diverse Ca^2+^-response elements (**CaRE**), whereas **Y_1,2,3…_** genes lacking Na/KRE are controlled by [Ca^2+^]_i_ only. The set of Na^+^_i_,K^+^_i_-sensitive transcription regulators shown as **X_n_**, **Y_n_** and **Z_n_** contributes to overall transcriptomic changes via activation of canonical response elements (**RE**) within **V_1,2,3…_** genes. Autocrine pathways triggered by the release of interleukin 6 and other [Na^+^]_i_/[K^+^]_i_-sensitive regulators of gene expression (**Z_m_**) may also contribute to overall transcriptomic changes via activation of their receptors (**7**).

The Na^+^_i_/K^+^_i_-dependent regulation of gene expression may have numerous physiological and pathological implications (Fig. 15). Here, we discuss a few of tissue-specific examples.

#### Excitation of neuronal cells

Almost 30 years ago, several research teams reported that expression of *Erg1* and other Na^+^_i_,K^+^_i_-sensitive IGR listed in Table 2 is strongly increased in neuronal cell subjected to excitation by diverse stimuli including neurotransmitters, depolarization and light [31]–[33]. The mechanisms of this phenomenon, widely employed for the identification of excited brain areas, remain largely unknown. It has been shown that activation of N-methyl-D-aspartate (NMDA) receptor contributes to elevation of *Egr1* mRNA content in excited neuronal cells. This link is so prominent that monitoring *Egr1* expression has been proposed as a biological assay for NMDA receptor activity. Given a key role of NMDA receptor activation in regulating synaptic strength, *Egr1* accumulation has also been connected with learning and memory (for review, see [34]). Indeed, experiments performed on *Egr1* deficient mice showed that their inability to form long-term memory in a variety of behavioural tasks [35]. It is generally accepted that *Egr1* expression in neuronal cells is triggered by [Ca^2+^]_i_ elevation [25]. However, ion currents through NMDA channels are mainly mediated by monovalent cations (P_Na_∼P_K_≫P_Ca_), and short periods of synaptic activity in apical dendrites and dendritic spines produce increases in [Na^+^]_i_, from ∼10 to 30 and 100 mM, respectively [36]. Recently, sharp elevation of [Na^+^]_i_ in response to local application of glutamate was demonstrated in neocortical neurons loaded with sodium-sensitive nanoprobe [37]. Here, we report that elevation of the [Na^+^]_i_/[K^+^]_i_ ratio in Ca^2+^-depleted HUVEC and HeLa cells augmented *Egr1* expression by ∼10-fold, which is comparable to increments detected in Ca^2+^-containing medium (Tables 4,5). In contrast, in RVSMC, *Egr1* up regulation was exclusively detected in the presence of Ca^2+^ (Table 6). Therefore, the relative impact of Ca^2+^_i_- mediated and Ca^2+^_i_ -independent mechanisms on Egr1 expression triggered by elevation of the [Na^+^]_i_/[K^+^]_i_ ratio in neuronal cells and their roles in memory formation and storage should be further examined in light of the data presented here.

#### Intensive exercise

Numerous studies demonstrated that the plasma concentration of IL6 increases up to 100-fold during muscular exercise. This increase is followed by the expression of the IL1 receptor agonist (*Il1ra*) and the anti-inflammatory cytokine IL10. Importantly, contracting skeletal muscle rather than the immune cells is the only source of the IL6 in circulation in response to exercise. The mechanism of this phenomenon, which plays a key role in the energy supply via elevation of glucose uptake in hepatocytes and lipolysis in adipose tissue, remains poorly understood (for comprehensive review, see [38]). It is known, however, that in both humans and experimental animals, intensive exercise increases [Na^+^]_i_ in skeletal muscles by 3–4-fold and decreases [K^+^]_i_ by 15–25% via activation of voltage-gated K^+^ and Na^+^ channels and partial inactivation of Na^+^,K^+^-ATPase [39], [40]. Here, we demonstrated that *Il6* is among the ubiquitous genes whose expression is strongly increased by elevation of the [Na^+^]_i_/[K^+^]_i_ ratio (Table 3). Therefore, our data allow us hypothesize that elevation of plasma level of IL6 during intensive exercise is caused by elevation of the [Na^+^]_i_/[K^+^]_i_ ratio in skeletal muscle that, in turn, increases expression of the [Na^+^]_i_/[K^+^]_i_-sensitive genes, including *Il6*.

#### Ischemia

**Figure 16:**
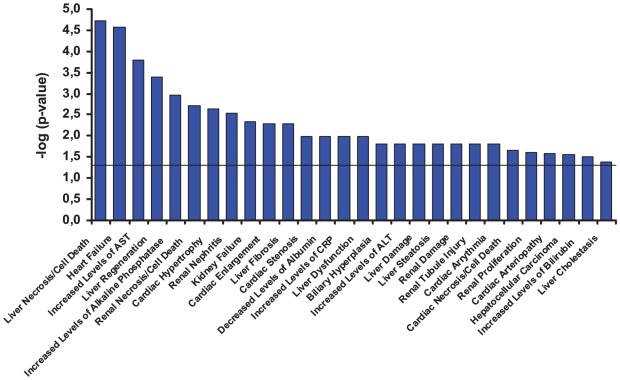
Disorders significantly associated with differential expression of ubiquitous Na^+^_i_,K^+^_i_-sensitive genes. The *p*-value and geometric fold change for each genes listed in Table 3 were imported into Ingenuity Pathway Analysis. The criteria with a threshold for significance of p = 0.05 (or 1.3 when expressed as -log(p-value) is shown by line.

To date, transcriptomic alterations in response to oxygen deprivation, such as modest hypoxia in solid tumours or in the adipose tissue of obese patients, have been largely ascribed to the overexpression of hypoxia-inducible factor 1 (HIF-1) known to be the molecular pO_2_ sensor that affects gene expression via its binding to *cis*-acting hypoxic-response elements (HRE) [41]. It should be noted, however, that the overwhelming number of genes, such as *Egr1, Atf3, Ptgs2, Il6, Ppp1r5, Hes1, Nfkbiz, Txnip, Adamts1, Egr 3, Cxcl2, Hsp70*, whose expression is strongly increased in cells subjected to ischemia/reperfusion both in vivo and in vitro [42]–[52], lack HRE. Significantly, we detected the above-listed genes among ubiquitous (*Egr1, Atf3, Ptgs2, Il6, Ppp1r5, Hes1, Nfkbiz, Txnip*, Tables 3 and 9) or cell-type specific (*Adamts1, Egr 3, Cxcl2, Hsp70*, Table 4,5,6) [Na^+^]_i_/[K^+^]_i_-sensitive genes. Since even transient ischemia increases [Na^+^]_i_ from 5–8 to 25–40 mM and causes reciprocal changes in [K^+^]_i_ [53], it can be hypothesized that inversion of the [Na^+^]_i_/[K^+^]_i_ ratio contributes to transcriptomic changes triggered by ischemia, with pathways that are complementary to those previously associated with activation of HIF-1. This conclusion is consistent with IPA Knowledge Base data showing abnormalities of gene expression revealed in ischemic liver, heart and kidney among disorders linked with ubiquitous Na^+^_i_/K^+^_i_-sensitive transcriptome (Fig. 16).

In conclusion, we report here that elevation of the [Na^+^]_i_/[K^+^]_i_ ratio affects expression of hundreds of genes via the activation of Ca^2+^-mediated and most importantly Ca^2+^-independent signaling pathways. The proposed hypothetical hierarchy of these pathways is depicted in Figure 15. Previously, we demonstrated that gain of Na^+^_i_ rather than loss of K^+^_i_ triggers augmented *c-Fos* expression in RVSMC [12]. We also reported that *c-Fos* expression triggered by [Na^+^]_i_ elevation occurs via Ca^2+^_i_-independent signaling mechanism that is not mediated by any known transcriptional elements in 5′-promoter [13]. We firmly believe that the newly gained knowledge of the Na^+^_i_/K^+^_i_-sensitive transcripts (genes X_1,2,3…_, Fig. 15) will allow researchers to take powerful bioinformatics approaches to identify previously-unfamiliar Ca^2+^_i_-independent mechanism of the excitation-transcription coupling as well as its involvement in cellular responses triggered by sustained elevation of the [Na^+^]_i_/[K^+^]_i_ ratio.

## Methods

### Cell cultures

We studied three cell types in the experiments reported here. RVSMC cells were isolated from rat aortae according to the procedures outlined in the *Guide for the Care and Use of Experimental Animals* endorsed by the Canadian Institutes of Health Research and accepted by the Institutional Animal Protection Committee of the CRCHUM. These cells maintain a number of characteristics of primary cultured RVSMC including high expression of smooth muscle-specific α-actin, SM22 protein and myosin light chain kinase [54]. The human cervical adenocarcinoma cell line HeLa was purchased from the American Type Culture Collection (Rockville, MA, USA). The human umbilical vein endothelial cells (HUVEC) were purchased from Lonza (Walkersville, MD, USA) and passaged 4–12 times. RVSMC and HeLa were maintained in Dulbecco's Modified Eagle Medium (DMEM, Invitrogen, Carlsbad, CA) supplemented with 10% fetal bovine serum (FBS) and 100 U/ml penicillin and 100 µg/ml streptomycin. HUVEC were cultured in complete endothelial cell growth medium-2 (EGM-2 BulletKit, CC3162, Lonza). All cell cultures were maintained in a humidified atmosphere with 5% CO_2_/balance air at 37°C. To establish quiescence, cells were incubated for 24 hr in the media in which concentration of FBS was reduced to 0.2%. The possible impact of Na^+^,K^+^-ATPase inhibition and Ca^2+^-depletion on cell viability and apoptosis was studied by lactate dehydrogenase (LDH) release and measurement of caspase-3 activity and chromatin cleavage, as described in details elsewhere [23], [24], [55].

### Elevation of the [Na^+^]_i_/[K^+^]_i_ ratio

Quiescent cells were washed with Ca^2+^- and K^+^-free DMEM (Sp-DMEM; Invitrogen, Carlsbad, CA) and incubated for 3 hr in either control medium containing 1.8 mM CaCl_2_ and 5 mM KCl (Sp-DMEM+Ca,K) or in Ca^2+^-free medium (Sp-DMEM+K) containing 50 µM EGTA and 10 µM BAPTA-AM. To increase the [Na^+^]_i_/[K^+^]_i_ ratio, the Na^+^,K^+^-ATPase activity was inhibited by the addition of ouabain or the omission of extracellular K^+^ (K^+^-free medium, Sp-DMEM+Ca). Because the affinity of the ubiquitous α1-Na^+^,K^+^-ATPase for ouabain and other cardiotonic steroids (CTS) in rodents is ∼1000-fold lower than in other mammalian species [56], ouabain was added to the media with human and rat cells at the concentrations of 3 and 3,000 µM, respectively.

### Intracellular content of exchangeable K^+^ and Na^+^

Intracellular content of exchangeable K^+^ and Na^+^ was measured as the steady-state distribution of extra- and intracellular ^86^Rb and ^22^Na, respectively. To establish isotope equilibrium, cells growing in 12-well plates were preincubated for 3 hr in control or K^+^-free medium (Sp-DMEM+Ca) containing 0.5 µCi/ml ^86^RbCl or 3 µCi/ml ^22^NaCl and ouabain was added for the next 3 hr. To test the action of K^+^-free medium, cells were washed twice with ice-cold Sp-DMEM+Ca. Then, cells loaded with ^22^Na were transferred to Sp-DMEM+Ca, containing 3 µCi/ml ^22^NaCl, whereas cells loaded with ^86^Rb were transferred to isotope-free Sp-DMEM+Ca. After 3 hr, the cells were transferred onto ice, washed 4 times with 2 ml of ice-cold medium W containing 100 mM MgCl_2_ and 10 mM HEPES-tris buffer (pH 7.4). The washing medium was aspirated and cells were lysed with 1% SDS and 4 mM EDTA solution. Radioactivity of incubation media and cell lysates was quantified, and intracellular cation content was calculated as *A/am*, where *A* was the radioactivity of the samples (cpm), *a* was the specific radioactivity of ^86^Rb (K^+^) and ^22^Na in the medium (cpm/nmol), and *m* was protein content. For more details, see [57].

### Measurement of intracellular Ca^2+^

Cells grown on glass cover slips were incubated for 30–40 min in medium containing 5 µM fura 2-AM, washed twice and kept for up to 30 min at room temperature before the experiments. Then, the cover slips treated as indicated in figure and table legends were mounted in a diagonal position in a 1×1 cm cuvette, and fluorescence was determined under permanent stirring at 37°C (λ_ex_ = 340 and 380 nm, slit 4 nm; λ_em_ = 510 nm, slit 12 nm), using a SPEX FluoroMax spectrofluorimeter (Edison, NJ). Free [Ca^2+^]_i_ was quantified as [Ca^2+^]_i_ = K_d_ (R−R_min_)x(R_max_−R)^−1^, where K_d_ is the dissociation constant of the Ca^2+^-fura 2 complex (224 nM at 37°C), and R = F_340_/F_380_ is the ratio of fluorescence at λ_ex_ = 340 and 380 nm. To determine F_max_, the cells were treated with 0.5 µM ionomycin in the presence of 1 mM CaCl_2_. To determine F_min_, MnCl_2_ was added at a final concentration of 2 mM.

### Measurement if intracellular BAPTA

HeLa cells seeded in 12-well plates were incubated for 3 hr in control or Ca^2+^-free media containing 2 µCi/ml [^14^C]-urea. Then, the cells were washed with 4×3 ml of ice-cold medium W and lysed for BAPTA measurement by addition of 200 µl of 10% trichloroacetic acid containing 1 mM CaCl_2_ or for radioactivity measurement as described above. The content of BAPTA in cell lysates was estimated by the increment of absorbance at 255 nm triggered by addition of 10 mM EGTA (*ΔA_255_*). Intracellular concentration of BAPTA (µmol/L) was calculated as *ΔA_255_/ΔA_255St_V_i_*, where *ΔA_255St_* is the Ca^2+^-dependent increment of A_255_ in the presence of 0.001 µmol BAPTA (internal standard) and *V_i_* is the volume of intracellular water (µl/mg protein). *V_i_* was calculated as the volume of [^14^C]-urea available space *V_i_ = V_o_A_i_/A_o_m*, where *A_i_* and *A_o_* are the radioactivity of [^14^C]-urea in the cell lysate and incubation medium, respectively (dpm), *m* is protein content in the cell lysate (mg), and *V_o_* is the volume of incubation medium (L) used for A_o_ determination.

### RNA isolation

Total RNA was extracted from cells grown in 6-well plates using TRIzol® reagent (Invitrogen, Carlsbad, CA and purified with the RNeasy® MinElute cleanup kit (Qiagen, Valencia, CA) following the manufacturers' protocols. Only the RNA samples that had more than 7.0 RNA integrity number (RIN) and no detectable genomic DNA contamination were used for the subsequent gene array analyses. RNA quality was assessed by 2100 Bioanalyzer (Agilent Technologies, Palo Alto, CA). Microarray experiments were performed with GeneChip® Human Gene 1.0 ST array (which detects 28,869 gene products) and GeneChip® Rat Gene 1.0 ST array (detects 27 342 gene products). On both arrays, each gene was represented by approximately 26 probes along the entire length of the transcript (Affymetrix, Santa Clara, CA). 100 ng of total RNA for each sample was processed with Ambion® WT Expression Kit (Invitrogen). This kit uses a reverse transcription priming method that specifically primes non-ribosomal RNA, including both poly(A) and non-poly(A) mRNA, and generates sense-strand cDNA as the final product. 5.5 µg of the single-stranded cDNA was fragmented and labeled using the Affymetrix GeneChip® WT Terminal Labeling Kit and 2.0 µg of the resulting cDNA was hybridized on the chip.

### GeneChip expression analysis

The whole hybridization procedure was conducted with the Affymetrix GeneChip® system according to the protocol recommended by the manufacturer. The hybridization results were evaluated with Affymetrix GeneChip® Command Console Software (AGCC). Quality of the chips was determined using Affymetrix Expression Console. Data analysis was performed within Partek Genomics Suite (Partek, St. Louis, Missouri). The data were initially normalized by Robust Multichip Average (RMA) algorithm, which uses background adjustment, quantile normalization and summarization. Then, normalized data were analyzed by principal component analysis (PCA) [18] to identify patterns in the dataset and high-light similarities and differences among the samples. Major sources of variability identified within the dataset by PCA were used as grouping variabilities for analysis of variance (ANOVA) with n = 4 for each group of samples. The ensuing data were filtered to identify transcripts with statistically significant variation of expression among the groups that are modulated by at least 20%, with multiple testing correction by the false discovery rate (FDR). The calculated *p*-value and geometric fold change for each probe set identifier were imported into Ingenuity Pathway Analysis (IPA, Ingenuity Systems, http://www.ingenuity.com) to ascertain networks, biological functions and their pathophysiological implications. Functional information on regulated gene was also obtained using PubMed and cited publications.

### Real-time quantitative RT-PCR

To validate data obtained by genome-wide Affymetric gene arrays, we estimated changes in the gene expression for several selected transcripts by qRT-PCR, which was performed using Express SYBR GreenER qPCR Supermix kit (Invitrogen, Carlsbad, CA, USA) according to the manufacture's instructions. The reaction was carried out with a 7900 HT Fast RT – PCR system (Applied Biosystems, Foster City, CA, USA). Primers for *Egr1, Ptgs2* and *Ppp1r15a* were designed using Primer3Plus online software from consensus sequences provided by Affymetrix for each gene of interest. The relevant primer sequences were: *Egr1*-sens (5′-TGACCGCAGAGTCTTTTCCT-3′), *Egr1*-anti-sense (5′-AGCGGCCAGTATAGGTGATG-3′), *Ptgs2*-sens (5′-TGTGTTGACATCCAGATCACAT-3′), *Prgs2*-anti-sense (5′-GCTGCTTTTTACCTTTGACACC-3′), *Ppp1r15a*-sens (5′-GGCCATCTATGTACCTGGAGA-3′) and *Ppp1r15a*-anti-sense (5′ GAGAAGCGCACCTTTCTGG-3′). All experiments were analyzed in duplicate. β_2_ microglobulin mRNA expression was used to normalize and compare the expression values of genes of interest. The results were quantified by the ΔΔCt method with Excel Microsoft software.

### Chemicals

Methyl-[^3^H]-thymidine was purchased from ICN Biomedicals, Inc. (Irvine, CA); ^22^NaCl and ^86^Rb were obtained from PerkinElmer (Waltham, MA); [^14^C]-urea was provided by Amersham (Montreal, PQ). DEVD-AMC, DEVD-CHO and z-VAD.fmk were procured from BIOMOL Research Laboratories (Plymouth Meeting, PA). The remaining chemicals were supplied by Gibco BRL (Gaithersburg, MO), Calbiochem (La Jolla, CA), Sigma (St. Louis, MO) and Anachemia (Montreal, QC).
